# Endotoxemia Is Associated with Altered Innate and Adaptive Immune Responses in Untreated HIV-1 Infected Individuals

**DOI:** 10.1371/journal.pone.0021275

**Published:** 2011-06-24

**Authors:** Anne Roslev Bukh, Jesper Melchjorsen, Rasmus Offersen, Jens Magnus Bernth Jensen, Lars Toft, Henrik Støvring, Lars Østergaard, Martin Tolstrup, Ole Schmeltz Søgaard

**Affiliations:** 1 Department of Infectious Diseases, Aarhus University Hospital Skejby, Aarhus, Denmark; 2 Department of Clinical Immunology, Aarhus University Hospital Skejby, Aarhus, Denmark; 3 Department of Biostatistics, Aarhus University, Aarhus, Denmark; University of Toronto, Canada

## Abstract

**Background:**

Microbial translocation may contribute to the immunopathogenesis in HIV infection. We investigated if microbial translocation and inflammation were associated with innate and adaptive immune responses in adults with HIV.

**Methodology/Principal Findings:**

This was an observational cohort study. Sera from HIV-infected and HIV-uninfected individuals were analyzed for microbial translocation (soluble CD14, lipopolysaccharides [LPS], endotoxin core antibody, and anti-α-galactosyl antibodies) and inflammatory markers (high sensitivity C-reactive protein, IL-6, IL-1 receptor antagonist, soluble tumor necrosis factor receptor II, and IL-10) with enzyme-linked immunosorbent assays. Peripheral blood mononuclear cells (PBMC) from HIV-infected persons and healthy controls (primed with single-stranded HIV-1-derived RNA) were stimulated with LPS, and cytokine production was measured. Finally, HIV-infected patients were immunized with Prevnar 7vPnC±CpG 7909 followed by Pneumo Novum PPV-23. Effects of microbial translocation and inflammation on immunization were analyzed in a predictive regression model. We included 96 HIV-infected individuals, 76 on highly active antiretroviral therapy (HAART), 20 HAART-naive, and 50 healthy controls. Microbial translocation and inflammatory markers were higher among HIV-infected persons than controls. Cytokine levels following LPS stimulation were increased in PBMCs from HAART-naive compared to HAART-treated HIV-infected persons. Further, RNA-priming of PBMCs from controls acted synergistically with LPS to augment cytokine responses. Finally, high serum LPS levels predicted poor vaccine responses among HAART-naive, but not among HAART-treated HIV-infected individuals.

**Conclusions/Significance:**

LPS acts synergistically with HIV RNA to stimulate innate immune responses *in vitro* and increasing serum LPS levels seem to predict poor antibody responses after vaccination among HAART-naive HIV-infected persons. Thus, our results suggest that microbial translocation may be associated with innate and adaptive immune dysfunction in untreated HIV infection.

## Introduction

Untreated HIV infection is characterized by progressive immune dysfunction. In the first weeks of infection, immense CD4+ T cell depletion and active viral replication occur, particularly in the intestinal mucosa [1], [2], [3]. This, in turn, leads to elevated levels of pro-inflammatory cytokines, increased apoptosis of epithelial cells, and altered tight junction protein composition [4] resulting in a functional degradation of the intestinal barrier [5]. These events are thought to induce microbial translocation, an enhanced transit of microbial products through intestinal mucosa to the blood stream.

Lipopolysaccharides (LPS) and LPS-related markers such as soluble CD14 (sCD14) have been used to quantify microbial translocation in peripheral blood [3], [6]. Anti-α-galactosyl (anti-Gal) immunoglobulins are potential novel markers that have not yet been tested in HIV patients [7]. Anti-Gal IgM and IgG interact specifically with the unique Galα1-3Galβ1-4GlcNAc-R epitope (α-galactosyl epitope) [8], which is found on the surface of cells of various organisms from prokaryotes to non-primate mammals [8], [9], [10]. Microbial translocation has also been observed in other clinical conditions including depression, burn injury, sepsis secondary to bacterial pneumonia, acute pancreatitis, and in excessive alcohol consumption [11], [12], [13], [14], [15]. Recently, North American and European cross sectional studies reported that LPS and sCD14 plasma levels, as well as immune activation, were increased among highly active antiretroviral therapy (HAART)-naive HIV-infected persons compared to HAART-treated HIV-infected and HIV-uninfected individuals [3], [16], [17], [18], [19], while in a Kenyan cohort both untreated and treated chronic HIV-infected subjects had significantly elevated levels of LPS [20]. Thus, microbial translocation may contribute to the persistent immune activation in HIV infection [3], [16], [17] and levels of LPS have been shown to correlate with disease severity in both HIV-1 and HIV-2 infections [21]. This hypothesis has been contradicted by findings from a longitudinal study of HIV-seroconverters in Uganda, which found no association between microbial translocation and HIV disease progression over an eight-year period [22]. Hence, the impact of microbial translocation on the immune system of people with HIV remains uncertain. Therefore, the objective of this study was to investigate if microbial translocation and inflammation were associated with innate and adaptive immune responses in adults with and without HIV.

## Methods

### Study design

This was an observational cohort study. Patients were recruited from a vaccination cohort consisting of otherwise healthy HIV-infected adults immunized with pneumococcal vaccines with or without CPG 7909 [23] (Text S1). HIV-uninfected controls were recruited from the blood bank at Aarhus University Hospital, Skejby, Denmark. Serum samples were stored at −80°C, while peripheral blood mononuclear cells (PBMCs) were stored at −170°C. The study was conducted at the Department of Infectious Diseases at Aarhus University Hospital, Skejby, Denmark. Written informed consent was obtained for all participants. Study protocols (Text S2) were approved by the Research Ethics Committee, Danish Data Protection Agency, and Danish Medicines Agency and registered at www.clinicaltrials.gov (NCT00562939). The methods and results from the main trial have been described elsewhere in details [23].

### Soluble markers of microbial translocation and pro-inflammation

Microbial translocation was investigated by quantification of sCD14, LPS, endotoxin core antibody IgG (endoCAb) (Hycult biotech), and anti-α-galactosyl antibodies (anti-Gal IgM and IgG) in serum samples. Inflammation was quantified by measuring serum levels of soluble tumor necrosis factor receptor II (sTNF-rII), interleukin-1 receptor antagonist (IL-1Ra), high sensitivity C-reactive protein (hs-CRP), interleukin-6 (IL-6), and interleukin-10 (IL-10) by enzyme-linked immunosorbent assay (ELISA) (RnD Systems and Invitrogen, DK). LPS levels were evaluated with a chromogenic limulus amebocyte lysate assay (LAL assay, QCL-1000^®^, Lonza, DK). The anti-Gal antibody assay was an in-house time-resolved immunofluorometric assay (TRIFMA). Briefly, plates were coated with 100 µL/well α-galactosyl-human serum albumin (α-Gal-HSA) glycoconjugates (Dextra, UK) or HSA (0.25 µg/mL), incubated in a humidifier over night at 4°C, and then blocked one hour with 1 mg HSA/mL tris-buffered saline (TBS). Next, 100 µL/well diluted serum samples, controls, blanks, and standard curve samples were added in duplicate. Following an over night incubation, biotinylated anti-human-IgG or –anti-human-IgM antibodies were added (100 µL/well, 0.2 µg/mL). After one hour, streptavidin-Eu was added to the plate. After an additional hour, Enhancement solution (PerkinElmer) was added. Counts per second were measured for each well on a Victor 3 Multilabel Reader (PerkinElmer). The relevant anti-HSA signal was subtracted from anti-α-Gal-HSA signals. Corrected signals were converted to measures of antibody concentration by relating them to a standard curve (the antibody concentration of standard curve samples with dilutions similar to samples were assigned a value of 100 AU). All other assays were performed according to the manufacturer's protocol. Up to three freeze-thaw cycles were performed on serum samples. Median intra-assay and inter-assay coefficients of variation for all markers were below 15%. All serum measurements were performed on pre-vaccinated samples.

### Responsiveness to LPS stimulation

The impact of HIV RNA and LPS on pro-inflammatory cytokine secretion was investigated in PBMCs from 20 HAART-treated and 20 HAART-naive individuals with HIV (with matched CD4+ cell count, age, and sex) with PBMCs exposed to one freeze-thaw cycle. For 48 hours, the PBMCs were either stimulated with 100 ng/mL LPS (Invivogen, France) or left untreated. A 25-plex cytokine luminex assay (Invitrogen, DK) was used to measure a large range of cytokines in harvested cell culture supernatants for instance TNF-α, IFN-α, and IFN-γ.

The co-stimulatory effect of viral RNA on LPS-induced cytokine production was further investigated in PBMCs from HIV-uninfected controls, which were exposed to one freeze-thaw cycle. PBMCs were either primed by transfection with increasing concentrations of HIV-1-derived single-stranded RNA (ssRNA40: 0, 0.01, 0.1, or 1.0 µg/mL) or unrelated control RNA (ssRNA41: 0.1 µg/mL) (both Invivogen, France). After incubation for 24 hours, PBMCs were stimulated with LPS at increasing concentrations (0, 1, 10, or 100 ng/mL). Supernatants were harvested and stored at −80°C. The pro-inflammatory marker TNF-α was measured in cell culture supernatants using a Cytoset ELISA (Invitrogen, DK).

### Adaptive immune response

All HIV-infected subjects were immunized, and all together three doses of vaccine were given. First with double the standard dose of 7-valent pneumococcal conjugated vaccine (7vPnC, Prevnar®, Wyeth) ±adjuvant (1 mg CpG 7909, Pfizer) at 0 and 3 months and with one single dose of 23-valent polysaccharide vaccine (PPV-23) (Pneumo Novum^®^, Sanofi-Pasteur MSD) ±1 mg CpG 7909 at 9 months [23]. Participants were also seen at 4 and 10 months for immunogenicity and safety follow-up.

Immune responses to pneumococcal vaccination were quantified by specific anticapsular IgG levels for all seven 7vPnC vaccine serotypes (4, 6B, 9V, 14, 18C, 19F, and 23F) [24] using a standardized ELISA by Statens Serum Institut, Denmark [25]. Serum samples were pre-adsorbed with optimal concentrations of pneumococcal cell wall and 22F capsular polysaccharides [26].

### Statistical analysis

HIV-infected individuals were categorized as “HAART-naive” if they had never been exposed to antiretroviral drugs or “HAART-treated” if, at inclusion, they received either a 3-drug regimen including a non-nucleoside reverse transcriptase inhibitor, a protease inhibitor, and/or abacavir or a 2-drug combination of a non-nucleoside reverse transcriptase inhibitor and a boosted protease inhibitor. All HAART-treated HIV-infected individuals had been treated for at least six months and had undetectable viral load at enrollment. HIV-uninfected persons were referred to as “controls”.

Medians and interquartile ranges (IQR) were calculated for all continuous variables. Sensitivity analyses were conducted to evaluate if participants, who on a single occasion demonstrated markers with extreme values, on their own caused the observed differences. Kruskal-Wallis rank test was used for comparison of more than two variables. Student's t-test was applied to test differences in the markers between groups. Logarithmic transformations (log_10_) were made when continuous outcomes did not follow a normal distribution. When logarithmic transformed values did not follow a normal distribution, Mann-Whitney rank sum was used. Spearman's correlation was used to evaluate correlations between markers.

LPS-responsiveness in PBMCs from HIV-infected individuals was evaluated by a stimulation index as the ratio between secreted cytokines from LPS-stimulated and untreated PBMCs. The measure of adaptive immune response was an aggregated outcome based on measurements of vaccine-specific antibodies (at 0, 3, 4, 9, and 10 months). To account for missing data, we employed a multiple imputation strategy [33] utilizing a monotone missing data pattern (no subjects re-entered the study after being lost to follow-up). This implied the model for imputations of an outcome could be based on values of the outcome at previous time points (3 withdrew consent, 4 were lost to follow-up, 1 was inappropriately enrolled, overall [23]). The effect of vaccination was estimated in a mixture model where current smoking status (yes/no), age, pre-vaccination CD4+ cell count, HIV RNA (log_10_), and TLR9 agonist-adjuvant (yes/no) in the pneumococcal vaccine were considered the fixed effects, whereas each individual was allowed a random intercept and random slope with respect to time. Correlation between the two random effects was allowed. Thus, individual random effects accounted for the variation between individuals in level and trend over time. For each marker, an estimate was calculated with a 95% confidence interval. The multivariate analysis was used to adjust for the known confounders. For all microbial translocation and inflammatory markers, we calculated both unadjusted and adjusted estimates (adjusted for current smoking status (yes/no), age, pre-vaccination CD4+ cell count, HIV RNA (log_10_), and TLR9 agonist-adjuvant (yes/no)). Analyses were stratified according to use of HAART at time of immunization. A significant estimate indicated the marker in question was able to predict the outcome of an antibody response. Stata 11.0 was used for all statistic analyses and the level of significance was set at 0.05.

## Results

The study included 96 HIV-infected individuals (20 HAART-naive and 76 HAART-treated) and 50 controls. Pre-vaccination characteristics and laboratory results for all subjects are shown in Table 1. No significant differences were found between HAART-treated and HAART-naive subjects in terms of CD4+ cell count, age, and sex. The median age of controls was lower than for HAART-treated and HAART-naive subjects.

**Table 1:** Characteristics and laboratory results for all participants pre-vaccination.

	HAART-treated (n = 76)	HAART-naive (n = 20)	Controls (n = 50)	*P* a
Age, years^b^	48.9 (42.8–59.9)	48.2 (40.6–55.3)	41.3 (31.3–51.3)	<0.0008
Sex, male, n (%)	65 (85.5)	16 (80)	30 (60)	= 0.004
BMI index^c^	23.5 (21.7–25.2)	24.6 (22.6–26.5)	-	= 0.19
CD4+ cell count, cells/µL	641 (489–837)	497 (373–812)	-	= 0.25
CD4 nadir, cells/µL	201 (71–243)	401 (313–659)	-	<0.0001
Log_10_ HIV RNA	1.60 (1.60–1.60)	4.33 (3.72–4.70)	-	<0.0001
Current smokers, n (%)	26 (34.2)	9 (45)	-	<0.001
sCD14, µg/mL	6.57 (4.85–9.39)	7.24 (5.07–11.4)	3.12 (2.76–3.66)	<0.0001
LPS, EU/mL	1.62 (1.28–2.07)	1.71 (1.49–1.98)	1.43 (1.14–1.88)	= 0.16
EndoCAb, GMU/mL	27.3 (15.4–51.7)	24.5 (17.1–36.5)	34.6 (25.4–56.9)	= 0.04
Anti-Gal^d^ IgM, AU^e^	178 (89.4–386)	140 (78.4–540)	152 (78.3–564)	= 0.83
Anti-Gal IgG, AU	9.28 (3.81–23.1)	9.30 (6.09–24.2)	13.38 (6.91–27.2)	= 0.30
sTNF-rII, ng/mL	5.0 (3.6–6.2)	5.8 (4.6–8.4)	4.3 (3.7–4.8)	<0.0002
IL-1Ra, pg/mL	66 (39–104)	88 (70–117)	67 (47–93)	= 0.044
hs-CRP, µg/mL	5.47 (2.18–13.0)	5.42 (1.79–8.15)	2.49 (1.00–7.52)	= 0.01
IL-6, pg/mL	0.32 (0.12–0.60)	0.44 (0.28–0.78)	0.13 (0.09–0.37)	= 0.006
IL-10, pg/mL	1.49 (0.77–2.27)	2.88 (1.79–3.87)	2.07 (1.13–3.86)	<0.001

a Global p-value, Kruskal-Wallis rank test.

b Unless otherwise indicated the median (IQR, interquartile range) is given.

c BMI, body mass index.

d Anti-Gal, anti-α-galactosyl.

e AU, arbitrary units.

### Microbial translocation in HIV-infected individuals

**Figure 1:**
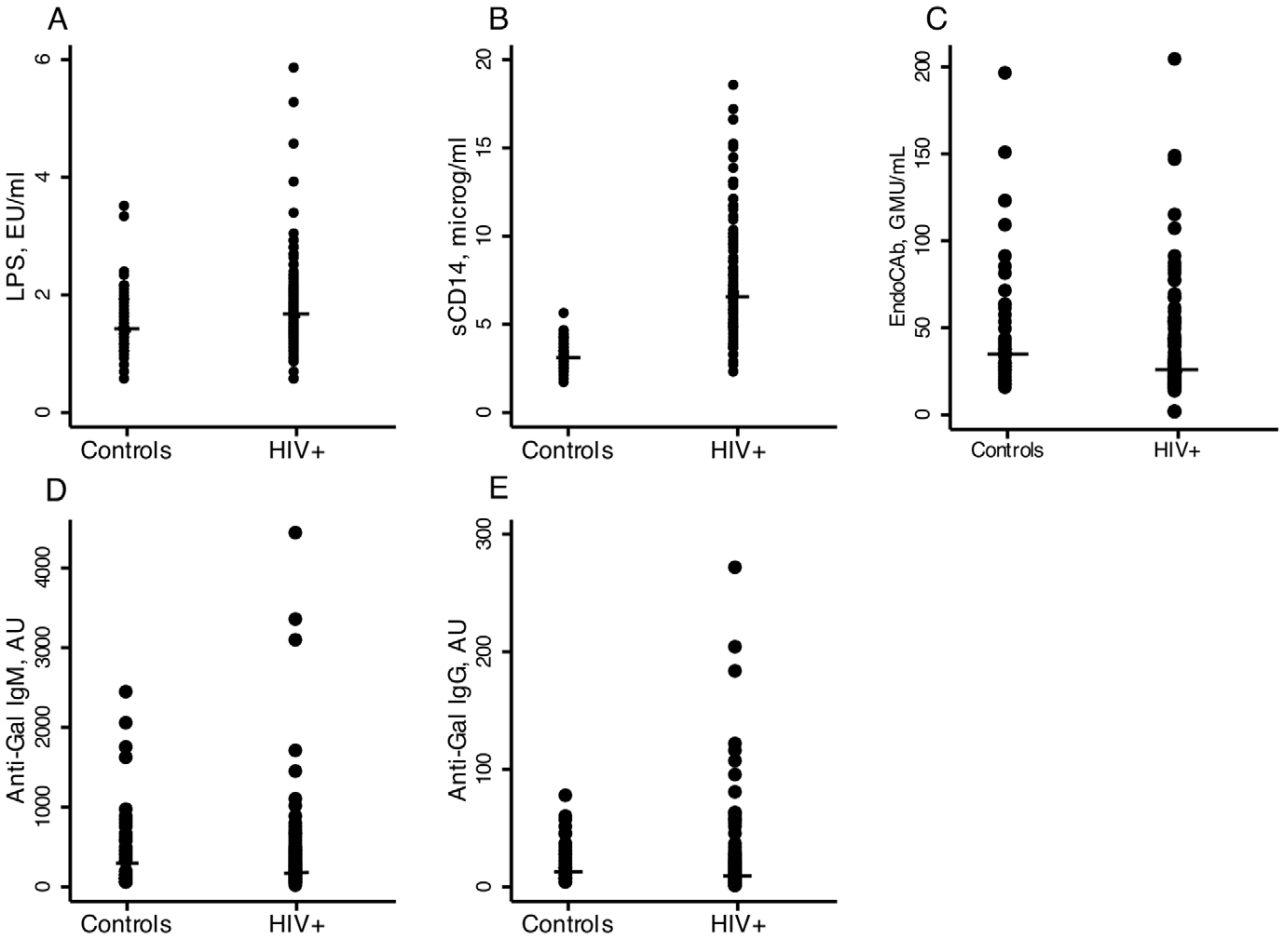
Microbial translocation in HIV-infected individuals and HIV-uninfected controls. (**A**) Median serum level of LPS in our cohort; controls: 1.55 EU/mL, HIV-infected persons: 1.82 EU/mL, p = 0.061. (**B**) Median serum level of sCD14 in our cohort; controls: 3.18 µg/mL, HIV-infected persons: 7.58 µg/mL, p<0.001. (**C**) Median serum level of endoCAb in our cohort; controls: 34.6 GMU/mL, HIV-infected persons: 26.2 GMU/mL, p = 0.08. (**D**) Median serum level of anti-Gal IgM in our cohort; controls: 288.4 AU, HIV-infected persons: 171.2 AU, p = 0.57. (**E**) Median serum level of anti-Gal IgG in our cohort; controls: 13.38 AU, HIV-infected persons: 9.28 AU, p = 0.14.

Soluble CD14 levels were significantly higher in HIV-infected subjects (6.58 µg/mL, IQR: 4.88–9.47) than in controls (3.12 µg/mL, IQR: 2.76–3.66, p<0.001), and there was a trend towards higher LPS in HIV-infected subjects (1.67 EU/mL, IQR: 1.30–2.06) compared to controls (1.43 EU/mL, IQR: 1.14–1.88, p = 0.06) (Fig. 1A and B). HIV-infected participants were stratified by use or non-use of HAART (Table 1). Between HIV-infected subjects and controls, no difference in endoCAb level was found (26.2 GMU/mL, IQR: 15.6–48.8 vs. 34.6 GMU/mL, IQR: 25.4–56.9, p = 0.08) (Fig. 1C). No difference was found in anti-Gal IgM levels between HIV-infected individuals (163 AU, IQR: 89.4–389) and controls (152 AU, IQR: 78.3–564, p = 0.57) (Fig. 1D) or in anti-Gal IgG levels between HIV-infected subjects and controls (9.28 AU, IQR: 4.00–23.1 vs. 13.4 AU, IQR: 6.91–27.2, p = 0.14) (Fig. 1E). No associations with HIV RNA, current or nadir CD4+ cell count were observed for either of the markers, and concentrations of sCD14, LPS, endoCAb, and anti-Gal IgM and IgG were comparable between HAART-treated and HAART-naive subjects. In addition, we found no significant associations between anti-Gal IgM and anti-Gal IgG with either sCD14 or LPS. An association was found between anti-Gal IgG and endoCAb for HIV-infected subjects, both HAART-naive and subjects on HAART treatment. No other associations were found between endoCAb and the other microbial translocation markers. Finally, sensitivity analyses revealed no impact of age on any of the microbial translocation markers.

### Elevated pro-inflammatory cytokine levels in HIV-infected individuals

The levels of the pro-inflammatory markers, sTNF-rII and IL-6, were significantly higher in HAART-treated and HAART-naive individuals compared with controls (Table 1). Compared with HAART-treated, HAART-naive subjects had higher serum levels of sTNF-rII (5.8 ng/mL, IQR: 4.6–8.4 vs. 5.0 ng/mL, IQR: 3.6–6.2, p = 0.03), IL-1Ra (88 pg/mL, IQR: 70–117 vs. 66 pg/mL, IQR: 39–104, p = 0.03), and IL-10 (2.9 pg/mL, IQR: 1.8–3.9 vs. 1.5 pg/mL, IQR: 0.8–2.3, p = 0.02). A near-significant correlation was observed between sCD14 and sTNF-rII in HIV-infected subjects (p = 0.053, rho = 0.20), but we found no associations between sCD14 and the other pro-inflammatory markers, or between LPS and any of the pro-inflammatory markers in either of the groups, except IL-10 in HAART-naive subjects (p<0.01, rho = −0.57). IL-10 showed a positive association with both sTNF-rII and IL-1Ra in HIV-infected subjects (sTNF-rII: p<0.001, rho = 0.35 and IL-1Ra: p<0.01, rho = 0.31). No significant associations were found between CD4+ cell counts or HIV RNA levels and any of the markers for pro-inflammation, except for IL-10, where all HIV-infected subjects had a negative association with CD4+ cell count (p = 0.013, rho = −0.25) and a positive correlation with HIV RNA level (p<0.01, rho = 0.28). Sensitivity analyses revealed no impact of age on any of the pro-inflammation markers, except for IL-10 in HAART-treated HIV-infected subjects, which showed a negative association between age and IL-10 (p = 0.04, rho = −0.24).

### *In vitro* LPS stimulation of HIV-infected PBMCs

**Figure 2:**
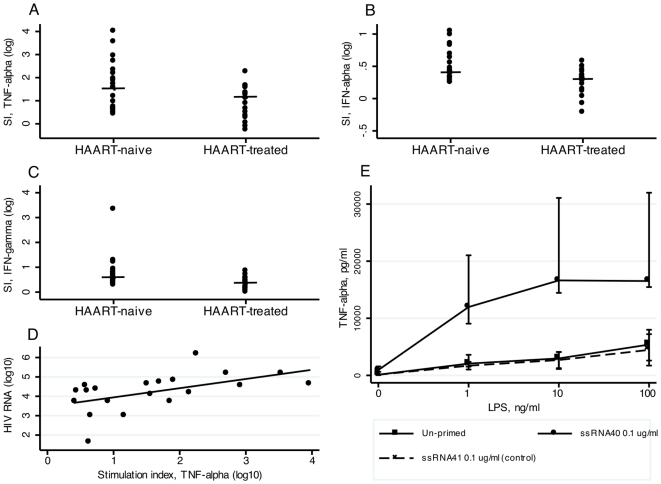
PBMC responsiveness to HIV RNA and LPS. (**A**) TNF-α stimulation index (SI) in HAART-naive and HAART-treated HIV-infected PBMCs stimulated with LPS (p = 0.03). (**B**) IFN-α SI in HAART-naive and HAART-treated HIV-infected PBMCs stimulated with LPS (p = 0.002). (**C**) IFN-γ SI in HAART-naive and HAART-treated HIV-infected PBMCs stimulated with LPS (p = 0.003). (**D**) TNF-α SI (log_10_) vs. HIV RNA (log_10_) in LPS-stimulated HAART-naive HIV-infected PBMCs, p<0.001. (**E**) Level of TNF-α in healthy PBMCs left untreated or primed with 0.1 µg/mL of ssRNA40 or ssRNA41 (control), and subsequently stimulated with LPS; unprimed vs. 0.1 µg/mL ssRNA in non-stimulated PBMCs, p<0.001, in PBMCs stimulated wth 1 ng/mL LPS, p<0.001, PBMCs stimulated with 10 ng/mL LPS, p<0.001, and PBMCs stimulated with 100 ng/mL LPS, p<0.001. Experiment was performed on PBMCs from four independent donors. Results depicted from one representative stimulation experiment, performed in triplicate.

Following LPS stimulation of PBMCs, greater stimulation indexes were observed in HAART-naive subjects compared with HAART-treated subjects for the main pro-inflammatory cytokines, TNF-α (p = 0.03), IFN-α (p = 0.002), and IFN-γ (p = 0.003) (Fig. 2A-C). We also observed significantly higher stimulation indexes for IL-1Ra (p = 0.004), IL-2R (p = 0.02), IL-5 (p = 0.01), IL-8 (p = 0.005), IL-13 (p = 0.02), and IP-10 (p = 0.04) in the HAART-naive individuals compared with HAART-treated subjects (data not shown). The HIV RNA and TNF-α stimulation indexes positively correlated in HAART-naive individuals (Fig. 2D), which suggested a dose-dependent relationship between HIV RNA and TNF-α produced by the LPS-stimulated PBMCs.

### Responsiveness of single-stranded RNA40-primed PBMCs to LPS *in vitro*

In PBMCs from HIV-uninfected controls, we found higher TNF-α responses in cells primed with HIV-1-derived single-stranded RNA (ssRNA40) (n = 4) compared with un-primed and unrelated ssRNA41-primed PBMCs after LPS stimulation (Fig. 2E). Both in non-LPS-stimulated and LPS-stimulated PBMCs we had significant results between unprimed and primed PBMCs (0, 1, 10, and 100 ng/mL LPS stimulated PBMCs, all p<0.001). TNF-α levels increased up to 100-fold after LPS stimulation (from 0 ng/mL LPS to 10 ng/mL LPS), which suggests a synergetic effect between LPS and HIV RNA. No additional increase in TNF-α production was observed by increasing LPS exposure from 10 to 100 ng/mL. Similar results were observed with other concentrations of ssRNA40 (0.01 and 1.0 µg/mL, Figure S1).

### High serum LPS levels predict poor adaptive immune responses in HAART-naive individuals

In HAART-naive subjects, LPS levels predicted the total specific IgG antibody response to pneumococcal vaccine (Table 2). The adjusted estimate for LPS was −2.62 (95% CI: −4.06–−1.17), which suggests LPS is an inverse, independent predictor of antibody response in untreated HIV-patients. No associations between vaccine responses and sCD14, anti-Gal immunoglobulins, or pro-inflammatory markers were found among HAART-naive subjects. In HAART-treated individuals, the unadjusted estimate for IL-6 was −0.35 (95% CI: −0.70–−0.01), while the adjusted estimate for IL-6 indicated no association with the vaccine response (p = 0.13). No other markers predicted antibody response in HAART-treated patients.

**Table 2:** Prediction of adaptive immune response in HAART-treated and HAART-naive HIV-infected individuals.

	HAART-treated (n = 76)		HAART-naive (n = 20)	
	Estimate, unadjusted	Estimate, adjusted^a^	Estimate, unadjusted	Estimate, adjusted
sCD14	−0.62 (−1.44–0.20)	−0.41 (−1.23–0.41)	−0.10 (−1.49–1.29)	−0.26 (−2.11–1.58)
LPS	0.51 (−0.37–1.39)	0.75 (−0.16–1.65)	−0.99 (−2.41–0.42)	−2.62 (−4.06–−1.17)
EndoCAb	0.002 (−0.003–0.007)	0.001 (−0.004–0.006)	−0.003 (−0.01–0.006)	−0.003 (−0.01–0.007)
Anti-Gal IgM	0.16 (−0.20–0.52)	0.08 (−0.28–0.43)	0.20 (−0.19–0.59)	0.46 (−0.13–1.04)
Anti-Gal IgG	−0.04 (−0.29–0.21)	−0.03 (−0.28–0.21)	−0.09 (−0.48–0.29)	−0.07 (−0.67–0.52)
sTNF-rII	0.05 (−0.79–0.89)	0.35 (−0.48–1.19)	−0.21 (−2.01–1.58)	0.05 (−2.33–2.42)
IL-1Ra	0.27 (−0.22–0.77)	0.20 (−0.29–0.69)	0.11 (−1.42–1.64)	1.04 (−1.04–3.12)
IL-6	−0.35 (−0.70–−0.01)	−0.28 (−0.65–0.08)	−0.33 (−0.87–0.22)	−0.18 (−0.88–0.51)
hs-CRP	−0.15 (−0.45–0.14)	−0.05 (−0.35–0.24)	−0.23 (−0.84–0.38)	−0.05 (−0.93–0.83)
IL-10	0.19 (−0.26–0.63)	0.20 (−0.25–0.65)	0.14 (−0.48–0.75)	0.32 (−0.54–1.17)

The predictive estimate of soluble markers for microbial translocation and pro-inflammation on adaptive immune response after pneumococcal vaccine in HAART-treated and HAART-naive HIV-infected individuals demonstrated by estimate and 95% confidence interval. LPS was found as an independent predictor after adjustment and mulitiple comparison.

a Adjustment for current smoking status, pre-vaccinated CD4+ cell count, age, HIV RNA (log_10_), and ±TLR9-agonist in the pneumococcal vaccine.

## Discussion

In this study, we evaluated the independent impact of microbial translocation and pro-inflammation on innate and adaptive immune responses. Interestingly, we found an inverse relation between baseline serum LPS and subsequent adaptive immune response in HAART-naive individuals. This association was not observed among HAART-treated subjects. We also found the release of pro-inflammatory cytokines after LPS stimulation was increased in PBMCs from viremic HAART-naive subjects compared to HAART-treated subjects, as well as in PBMCs from healthy controls pre-treated with HIV-1-derived RNA, and similar findings have been demonstrated in previous studies [20], [27]. Further, the TNF-α response depended more on increasing HIV RNA levels than on changes in LPS level. Thus, LPS may act in synergy with HIV RNA and cause a disruption of adaptive immune functions by inappropriate immune diversion.

Anti-Gal immunoglobulins are potential novel markers for microbial translocation and HIV infection. Specific anti-Gal antibodies make up approximately 1% of circulating IgG antibodies [8], [28]. We did not however observe any differences in concentrations of anti-Gal antibodies between HIV-infected individuals and controls. One possible caveat is that we did not have information about the subjects' ABO-group statuses, which may have influenced the results [29], since it is possible with interference due to cross-activity between B antigen and α-Gal antigen. The lack of differences may also have resulted from compromised adaptive immune responses due to HIV-infection where additional antibodies cannot be produced, although the load of α-galactosyl epitopes may be increased. The mechanisms are not fully established and further investigations are needed before anti-Gal immunoglobulins can be used as markers of microbial translocation in HIV-infected subjects. Thus, serum concentration production of anti-Gal immunoglobulins seems unaffected by HIV-infection, but further studies must be conducted before it is certain how anti-Gal immunoglobulins act in HIV-infected individuals.

We did not retrieve any association between endoCAb and LPS or endoCAb and sCD14, while another study found an inverse correlation to LPS [16]. As sCD14 and anti-Gal immunoglobulins, endoCAb is produced by the host and therefore in need of a functional immune system. We found a positive correlation between anti-Gal IgG and endoCAb, and since both are class IgG and produced in response to foreign microbial product, it is expectable.

This study had some limitations. A cross sectional study design has obvious limitations due to lack of follow up. Participants in our HAART-naive group consisted of a relatively small number compared to other studies on microbial translocation [3], [18] though even smaller number of HAART-naive patients is seen [30]. The strength of the results may be lower because of the small number of patients in the HAART-naive group. Therefore it would be relevant to enroll more HAART-naive patients in future studies and include a follow up. We did not find any significant difference between HAART-treated and HAART-naive individuals' level of LPS in contrast to other studies [3], [19], but we did find a trend toward difference between uninfected and HIV-infected individuals.

No significant association was found between LPS and sCD14 in our study, which is in accordance with findings of some studies [22] while contradicting findings from other studies [3]. The reason for these discrepancies is not clear. However, sCD14 inhibits cell responses to LPS by diverting LPS away from membrane-bound CD14 and by promoting LPS efflux from cell surface and transferring it to plasma lipoproteins [31], [32]. Therefore, less LPS may be present for detection in serum samples because of the influence of larger amounts of sCD14.

LPS is the only primary marker of microbial translocation measured in our study, since sCD14 and endoCAb are host markers. Another primary marker, which might be relevant to measure, could be bacterial ribosomal 16S RNA (16S rDNA), which is found higher in HIV-infected individuals compared to uninfected, and 16S rDNA correlates with LPS [18]. However, there have been some discrepancies about the value of this marker [33].

In this study we used samples from a vaccination cohort. Protocol samples were collected at three immunizations and at follow-up [23], which allowed us to study the association of pre-vaccination markers with adaptive immune responses prospectively. The cytokine response to LPS stimulation in PBMCs from HIV-infected subjects suggested a connection between in vivo HIV plasma RNA levels and the ex vivo magnitude of pro-inflammatory response. In addition, PBMCs from healthy controls primed with HIV-1-derived RNA displayed a TNF-α response that was independent of increases in LPS concentration. Thus, TNF-α production appears to be amplified by the presence of HIV RNA and LPS and is consistent with findings of recent studies [20], [27], which likewise demonstrated that LPS stimulation of HIV-infected PBMCs revealed a significant increase in TNF-α level compared to uninfected PBMCs. Collectively, our findings suggest increasing levels of viral RNA enhance the susceptibility of PBMCs to LPS stimulation and, thereby, further activate the immune system.

The innate toll-like receptors (TLRs) recognize pathogens and, upon activation, induce and direct immune responses. HIV single-stranded RNA, a TLR7- and TLR8-ligand [34], triggers initiation of viral transcription and subsequent HIV replication in dendritic cells through TLR8 [35]. Redundancies in TLR expression and regulation have been demonstrated with TLR4 and TLR8 [36], [37]. This illustrates crosstalk between TLRs; with TLRs sensitized to their cognate ligands resulting in further enhanced pro-inflammatory cytokine responses, which contributes to persistent immune activation. TLR crosstalk is present in other conditions such as complement-TLR crosstalk, where one study suggests that pathogens exploit this pathway besides undermining complement and TLRs [38], and another study demonstrated that TLR crosstalk controls B cell responses on several levels, and the outcome of TLR crosstalk in B cells from patients with periodontal diseases and diabetes are influenced by disease pathology [39]. Enhanced expression of TLRs and increased responsiveness to their ligands, such as LPS in HIV-infection [37], may play an important role in adaptive immune responses; TLRs appear to be essential in the initiation of adaptive immune responses [40]. Normalization of TLR levels is observed after viral suppression by HAART [37], which is consistent with our findings that lower cytokine levels were produced by PBMCs from HAART-treated subjects compared with HAART-naive patients. Concurrently, in HAART-treated individuals, adaptive immune responses were not predicted by LPS, thereby indicating a positive effect of treatment on both innate and adaptive immunity.

Pneumonia is more common in HIV-infected individuals with a 6-fold higher incidence than in HIV-uninfected individuals [41] leading to excess morbidity and mortality [41], [42]. Immune responses to immunization in HIV-populations appear superior in subjects on HAART with suppressed HIV RNA compared to viremic HAART-naive HIV-infected subjects [43], [44]. In accordance with our results, the effect of immunization appear greater in HIV-infected subjects on HAART.

It is important to further investigate pneumococcal vaccination effectiveness in HIV-infected subjects in relation to microbial translocation and innate immune function. If our findings translate in to clinical effectiveness, then increased LPS levels at the time of immunization would independently predict vaccine failure in HAART naive individuals.

There is a great deal of uncertainty regarding the relationship between HIV disease progression and microbial translocation and subsequent inflammatory immune responses [22]. Though antibody response is not a clinical endpoint, a recent study showed vaccination responses following hepatitis B immunization predicted HIV disease progression (Landrum et al., at Conference on retroviruses and opportunistic infections, 2010∶625). In need of clinical endpoints, adaptive immune responses may thus be viewed as a surrogate marker of HIV disease progression. However, it should be noted that due to the above-mentioned limitations and to the study design, our findings could not establish a causal link between microbial translocation and immune activation and HIV disease progression.

In conclusion, we found LPS to be an independent predictor of adaptive immune response in untreated HIV-infected individuals. Our results suggest HIV RNA and LPS act in synergy and that their concerted action brings about increased cytokine responsiveness through innate immune recognition pathways. By reducing viremia, HAART treatment increases the tolerance to LPS, which in turn reduces further immune activation. Hence, earlier initiation of HAART may offset the immune disruption caused by microbial translocation and should be investigated in future studies.

## Supporting Information

Figure S1 PBMC responsiveness to HIV RNA and LPS. (**A**) Level of TNF-α in healthy PBMCs left untreated or primed with 0.01 µg/mL of ssRNA40 or ssRNA41 (control), and subsequently stimulated with LPS; unprimed vs. 0.01 µg/mL ssRNA in unstimulated PBMCs (p = 0.13), in PBMCs stimulated wth 1 ng/mL LPS (p = 0.32), PBMCs stimulated with 10 ng/mL LPS (p = 0.14), and PBMCs stimulated with 100 ng/mL LPS (p = 0.32). (**B**) Level of TNF-α in healthy PBMCs left untreated or primed with 1.0 µg/mL of ssRNA40 or ssRNA41 (control), and subsequently stimulated with LPS; unprimed vs. 1.0 µg/mL ssRNA in unstimulated PBMCs (p<0.0001), in PBMCs stimulated wth 1 ng/mL LPS (p<0.0001), PBMCs stimulated with 10 ng/mL LPS (p<0.001), and PBMCs stimulated with 100 ng/mL LPS (p<0.002). Both experiments were performed on PBMCs from four independent donors. Results depicted from one representative stimulation experiment, performed in triplicate.
(TIF)

Text S1 Flowchart from main trial.
(PDF)

Text S2 Pneumococcal CPG7909 protocol from main trial.
(DOC)
