# Supplementary figures and images for: Endotoxemia Is Associated with Altered Innate and Adaptive Immune Responses in Untreated HIV-1 Infected Individuals
Source: PLoS One. 2011 Jun 24;6(6):e21275. doi: 10.1371/journal.pone.0021275 (PMC3123300; doi:10.1371/journal.pone.0021275)

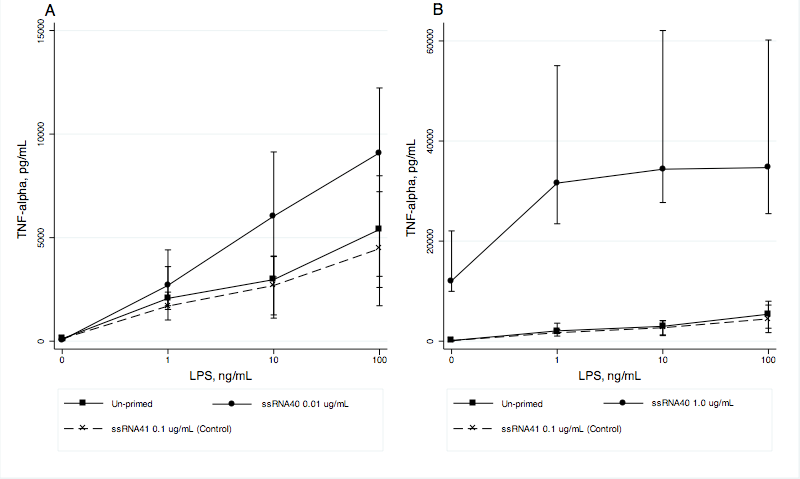

Supplement: Figure S1 — PBMC responsiveness to HIV RNA and LPS. (A) Level of TNF-α in healthy PBMCs left untreated or primed with 0.01 µg/mL of ssRNA40 or ssRNA41 (control), and subsequently stimulated with LPS; unprimed vs. 0.01 µg/mL ssRNA in unstimulated PBMCs (p = 0.13), in PBMCs stimulated wth 1 ng/mL LPS (p = 0.32), PBMCs stimulated with 10 ng/mL LPS (p = 0.14), and PBMCs stimulated with 100 ng/mL LPS (p = 0.32). (B) Level of TNF-α in healthy PBMCs left untreated or primed with 1.0 µg/mL of ssRNA40 or ssRNA41 (control), and subsequently stimulated with LPS; unprimed vs. 1.0 µg/mL ssRNA in unstimulated PBMCs (p<0.0001), in PBMCs stimulated wth 1 ng/mL LPS (p<0.0001), PBMCs stimulated with 10 ng/mL LPS (p<0.001), and PBMCs stimulated with 100 ng/mL LPS (p<0.002). Both experiments were performed on PBMCs from four independent donors. Results depicted from one representative stimulation experiment, performed in triplicate. (TIF) [file pone.0021275.s001.tif]
