# Supplementary material for: Endotoxemia Is Associated with Altered Innate and Adaptive Immune Responses in Untreated HIV-1 Infected Individuals
Source: PLoS One. 2011 Jun 24;6(6):e21275. doi: 10.1371/journal.pone.0021275 (PMC3123300; doi:10.1371/journal.pone.0021275)
Supplement: Text S1 — Flowchart from main trial. (PDF) [file pone.0021275.s002.pdf]

Flow chart for main trial

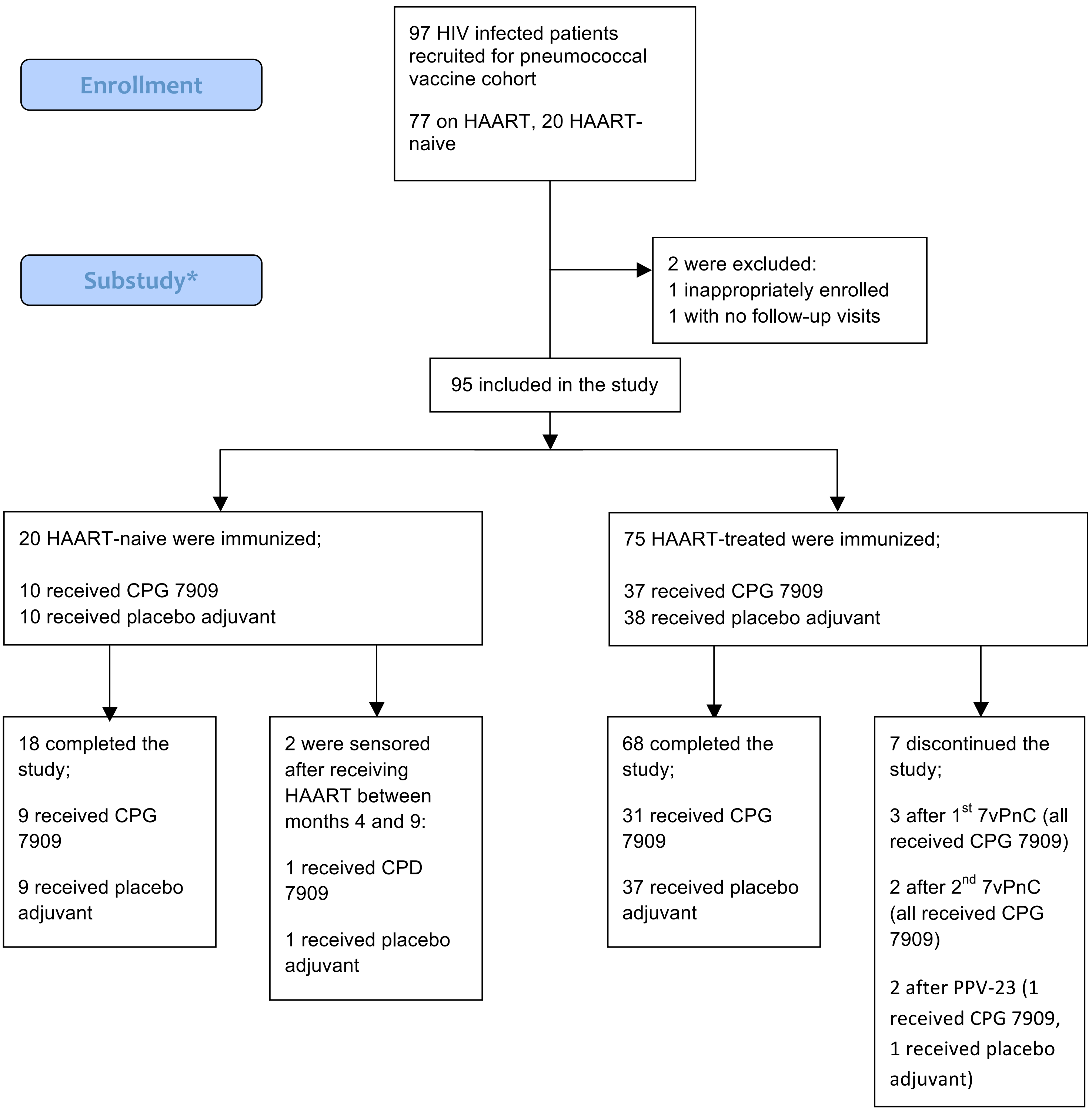

*In the substudy "Microbial translocation is associated with altered innate and adaptive immune responses in untreated HIV-infected individuals" 20 HAART-naive and 76 HAART-treated HIV-infected individuals were included and pre-vaccinated serum samples were used for the assays.
