# Supplementary material for: Endotoxemia Is Associated with Altered Innate and Adaptive Immune Responses in Untreated HIV-1 Infected Individuals
Source: PLoS One. 2011 Jun 24;6(6):e21275. doi: 10.1371/journal.pone.0021275 (PMC3123300; doi:10.1371/journal.pone.0021275)
Supplement: Text S2 — Pneumococcal CPG7909 protocol from main trial. (DOC) [file pone.0021275.s003.doc]

**Immune Response to** Toll-like receptor 9-agonist Adjuvanted **Pneumococcal Vaccination in HIV Infected Adults**

**Sponsor:**

**Lars Østergaard; MD, PhD, DMSc, Medical director, A/Prof.,**

**Department of Infectious Diseases**

**Skejby hospital, Aarhus university Hospital**

**Brendstrupgaardvej**

**8200 Aarhus N**

**Study vaccines**: Pneumo Novum®, Sanofi Pasteur MSD

Prevenar®, Wyeth

Combined with 1 mg CPG 7909 formulated in 100 μl PBS buffer, Coley Pharmaceutical Group or placebo (100 μl) according to randomization.

**EudraCT number:** 2007-001588-31

**Protocol code:** OSS0001

**Date of approval:**

**Title:** **Immune Response to** Toll-like receptor 9-agonist adjuvanted **Pneumococcal Vaccination in HIV Infected Adults.**

**Co-ordinating author:** Ole Schmeltz Søgaard, MD, Department of infectious diseases, Skejby hospital – Aarhus university hospital

**Contributing authors:** Lars Østergaard; MD, PhD, DMSc, Medical director, A/Prof., Department of infectious diseases, Skejby hospital – Aarhus university hospital

Henrik Carl Schönheyder, MD, PhD, DMSc, Professor, Department of Clinical Microbiology, Aalborg Hospital – Aarhus University Hospital, Mølleparkvej 8 A ,9100 Aalborg, Denmark

Nicolai Lohse, MD, PhD, Department of Clinical Epidemiology, Aarhus University Hospital, Ole Worms Allé 1150, 8000 Aarhus C

**Sponsor signatory:** Lars Østergaard**;** MD, PhD, DMSc, Medical director, A/Prof., Department of infectious diseases, Skejby hospital – Aarhus university hospital, Brendstrupgaardvej, 8200 Aarhus N, tel: 89498300 fax: 89498490 email: oes@sks.aaa.dk Denmark

**Principal investigator:** Ole Schmeltz Søgaard, MD, Department of infectious diseases, Skejby hospital – Aarhus university hospital, Brendstrupgaardvej, 8200 Aarhus N, Denmark, tel: 89498492 fax: 89498490 email: oks@sks.aaa.dk

**Co-investigator:** Lars Østergaard**;** MD, PhD, DMSc, Medical director, A/Prof., Department of infectious diseases, Skejby hospital – Aarhus university hospital, Brendstrupgaardvej, 8200 Aarhus N, tel: 89498300 fax: 89498490 email: oes@sks.aaa.dk Denmark

**Collaborating laboratories:** Statens Serum Institute, Artillerivej 5 2300 Kbh S, Denmark

Flow Applications, Inc. / 300 E. High St. / Okawville, IL 62281, USA

Department of Clinical Biochemistry, Skejby Hospital – Aarhus university hospital, Brendstrupgaardvej, 8200 Aarhus N, Denmark

Department of Clinical Biochemistry, Aarhus Hospital – Aarhus university hospital, Nørrebrogade, 8000 Aarhus C, Denmark

Hospital Pharmacy Aarhus, Central Region Denmark, Aarhus Universitetshospital, Aarhus Sygehus, Nørrebrogade 44
8000 Århus C, Telephone: +45 8949 3610, Denmark

**Study monitor:** The GCP-unit, Oluf Palmes Allé 15, 8200 Aarhus N, Denmark,

Tel 87284380

**Conduct of study:** The study is conducted according to Good Clinical Practice guidelines, the 1996 version of the Declaration of Helsinki and Danish ethical and legal authority regulations.

**Time schedule:** The first participant is expected to be enrolled in the study on the 1^st^ of October 2007. The last participant is expected to be enrolled no later than the 1^st^ of April 2008 and last follow-up will be on the 1^st^ of October 2008.

TABLE OF CONTENTS:

[LIST OF ABBREVIATIONS 6](#__RefHeading___Toc169490144)

[BACKGROUND: 7](#__RefHeading___Toc169490145)

[PROTOCOL 13](#__RefHeading___Toc169490146)

[OBJECTIVES: 13](#__RefHeading___Toc169490147)

[MAIN ASSESSMENT PARAMETERS: 13](#__RefHeading___Toc169490148)

[ENROLMENT: Randomization; 15](#__RefHeading___Toc169490149)

[SUBJECT WITHDRAWAL FROM THE STUDY: 15](#__RefHeading___Toc169490150)

[SUBJECT WITHDRAWAL FROM INVESTIGATIONAL PRODUCT 16](#__RefHeading___Toc169490151)

[DATA TO BE INCLUDED IN THE CASE REPORT FORM: 16](#__RefHeading___Toc169490152)

[REASONS FOR “BREAKING” A PARTICIPANTS RANDOMIZATION CODE: 17](#__RefHeading___Toc169490153)

[SPONSOR’S TERMINATION OF STUDY: 17](#__RefHeading___Toc169490154)

[PARTICIPANT INCLUSION CRITERIA: 17](#__RefHeading___Toc169490155)

[PARTICIPANT EXCLUSION CRITERIA: 17](#__RefHeading___Toc169490156)

[PROCEDURES: 17](#__RefHeading___Toc169490157)

[VACCINES AND CONTROL INJECTIONS: 18](#__RefHeading___Toc169490158)

[PRIMARY EFFICACY PARAMETER AND ANALYSIS OF ANTIBODY RESPONSE 19](#__RefHeading___Toc169490159)

[SECONDARY EFFICACY PARAMETER AND ANALYSIS OF ANTIBODY RESPONSE 19](#__RefHeading___Toc169490160)

[PNEUMOCOCCAL CARRIAGE 20](#__RefHeading___Toc169490161)

[SAFETY/TOLERABILITY ASSESSMENT PARAMETERS: 20](#__RefHeading___Toc169490162)

[ADVERSE EVENTS (AEs): 20](#__RefHeading___Toc169490163)

[SERIOUS ADVERSE EVENT (SAE) DEFINITION: 20](#__RefHeading___Toc169490164)

[SUSPECTED UNEXPECTED SERIOUS ADVERSE EVENT REACTION (SUSAR) DEFINITION: 21](#__RefHeading___Toc169490165)

[SUBMITTING SUSPECTED UNEXPECTED SERIOUS ADVERSE EVENT REACTION (SUSAR) REPORTS 21](#__RefHeading___Toc169490166)

[FOLLOW-UP OF ADVERSE EVENTS AND SERIOUS ADVERSE EVENTS AND ASSESSMENT OF OUTCOME 22](#__RefHeading___Toc169490167)

[TREATMENT OF ADVERSE EVENTS 22](#__RefHeading___Toc169490168)

[SAFETY UPDATES 22](#__RefHeading___Toc169490169)

[SOLICITED ADVERSE EVENTS 24](#__RefHeading___Toc169490170)

[EVALUATING ADVERSE EVENTS AND SERIOUS ADVERSE EVENTS 24](#__RefHeading___Toc169490171)

[ASSESSMENT OF INTENSITY 24](#__RefHeading___Toc169490172)

[DATA EVALUATION: CRITERIA FOR EVALUATION OF OBJECTIVES **Fejl! Bogmærke er ikke defineret.**](#__RefHeading___Toc169490173)

[ESTIMATED SAMPLE SIZE 27](#__RefHeading___Toc169490174)

[ACCESS TO CRFs, PARTICIPANT RECORD AND STUDY RELATED DOCUMENTS 29](#__RefHeading___Toc169490175)

[STUDY QUALITY CONTROL AND ASSESSMENT 29](#__RefHeading___Toc169490176)

[ETHICAL CONSIDERATIONS 29](#__RefHeading___Toc169490177)

[WRITTEN CONSENT AND PARTICIPANT INFORMATION 30](#__RefHeading___Toc169490178)

[DATA HANDLING AND MANAGEMENT 30](#__RefHeading___Toc169490179)

[FINANCING 30](#__RefHeading___Toc169490180)

[PARTICIPANT INSURANCE 31](#__RefHeading___Toc169490181)

[TRIAL REGISTRATION 31](#__RefHeading___Toc169490182)

[PUBLICATION OF STUDY RESULTS 31](#__RefHeading___Toc169490183)

[PROTOCOL SYNOPSIS 31](#__RefHeading___Toc169490184)

[APPENDIX LIST 32](#__RefHeading___Toc169490185)

[SCHEDULE OF ASSESSMENTS/PROCEDURES 33](#__RefHeading___Toc169490186)

[REFERENCE LIST 34](#__RefHeading___Toc169490187)

# LIST OF ABBREVIATIONS

**AE** Adverse event

**AIDS** Acquired Immune Deficiency Syndrome

**CD4** Cluster of Differentiation 4

**CD4 count** CD4-positive cell count - a surrogate marker of immune status in HIV-infected persons

**CIOMS form** Council for International Organizations of Medical Sciences

**CONSORT** Consolidated Standards of Reporting Trials

**CpG** Cytosine and Guanine separated by a phosphate

**CPG 7909** A synthetic TLR9-agonist manufactured by Coley Pharmaceutical Group Inc.

**CRF** Case Report Form

**FDA** Food and Drug Administration

**HAART** Highly Active AntiRetroviral Therapy

**HIV** Human Immunodeficiency Virus

**IL-2** Interleukin 2

**ODN** OligoDeoxyNucleotides

**OPA** OpsonoPhagocytic Activity

**SAE** Serious Adverse Event

**TLR9** Toll-Like Receptor 9

# BACKGROUND:

With the advent of Highly Active AntiRetroviral Therapy (HAART), marked reductions in AIDS-associated opportunistic infections and mortality have occured. Nonetheless, in the HAART era, the incidence of pneumococcal pneumonia and invasive pneumococcal disease remains 15-40 times higher in HIV-infected vs non-HIV infected persons and pneumococcal disease is a major source of morbidity and mortality among HIV-infected individuals {{8 McEllistrem,M.C. 2002;7 Nuorti,J.P. 2000; 6 Redd,S.C. 1990; 9 Barry,P.M. 2006; 10 Grau,I. 2005; }}. Vaccination is the primary available tool for preventing invasive pneumococcal diseases. Thus effective pneumococcal vaccination could potentially reduce morbidity and mortality in HIV-infected individuals.

Two types of pneumococcal vaccines are registered for use in humans. The 23-valent polysaccharide vaccine covers approximately 70% of the serotypes causing pneumococcal pneumonias. Antibody response in HIV-infected children and adults using polysaccharide pneumococcal vaccines has been examined in a number of studies. Among children and adults receiving HAART, polysaccharide vaccine (i.e. Pneumo Novum®, Sanofi Pasteur MSD) elicits only modest increases in serotype specific antibody concentrations, antibody titers wane quickly, functional antibody responses are lower than in healthy controls, and effects on rates of pneumococcal disease are inconsistent {{12 Feikin,D.R. 2004;13 Feikin,D.R. 2004; 26 Rodriguez-Barradas,M.C. 1996; 30 Rodriguez-Barradas,M.C. 2003; 37 Kroon,F.P. 1999;136 Nielsen,H. 1998; }}.

The reason for the poor immunogenicity in immune deficient individuals is thought to be the T-cell independent nature of the polysaccharide antigen, which is able to stimulate mature B-cell without the help of T-cells. In humans, the B-cells of the majority of immune deficient individuals (i.e. HIV infected) respond inadequately to most of the polysaccharide antigens. Furthermore, the absence of a T-cell immune response prevents B-cell maturation, as well as the induction of B-cell immunological memory and the associated affinity/avidity maturation of antibodies. Thus, the antibodies elicited by the polysaccharide vaccine disappear quickly.

The newer 7-valent conjugated pneumococcal vaccine (Prevenar) was developed for use in infants and toddlers. Despite the childrens immature immune system the vaccine is immunogenic due to its Th2-dependent immune response. The safety and efficacy of Prevenar has been proven to be excellent and the vaccine is now part of the immunization program for children in several countries including USA, England and Holland and was in March 2007 recommended by WHO to be included in all national immunization programs{{138 Anonymous 2007;1 Anonymous 2007; }}.

Conjugated pneumococcal vaccines were expected to be well suited for immunocompromised individuals and they have been tested in a number of trials in adults. However, a single immunization with a conjugated pneumococcal vaccine has not been demonstrated to be superior to the polysaccharide vaccine{{12 Feikin,D.R. 2004;13 Feikin,D.R. 2004; 26 Rodriguez-Barradas,M.C. 1996; 30 Rodriguez-Barradas,M.C. 2003;36 Kroon,F.P. 2000; }}. There are several possible explanations for these findings: 1. The antigenic content of the polysaccharide vaccine is higher than in any of the conjugate vaccines (for example, 25 μg of each of 23 serotypes in Pneumo Novum versus 2 μg of six of the seven serotypes in Prevenar with the exception of 4 μg of serotype 6B). 2. The methodologies of the serologic assays in the published studies were all different, leading to uncertain interpretation of immunogenicity results. 3. The adult populations studied were composed of diverse risk groups, with presumably differing levels of immunocompetence. Immune responsiveness to conjugate and polysaccharide vaccines may vary in different groups, and results obtained in healthy 50-year-olds may not reflect what is seen in higher risk elderly subjects. 4. The design of the trials were widely variant in key points such as dosing, administration, confounder control and populations. 5. Vaccine formulations varied greatly between studies{{140 Abraham-Van Parijs,B. 2004; }}.

A recent dose-ranging trial with conjugate pneumococcal vaccine in seniors (70+) found that a double dose of Prevenar (1.0 ml) was significantly more immunogenic than a single dose (0.5 ml). A dosing of 2.0 ml (four doses of Prevenar) was not more immunogenic than a dosing of 1.0 ml. Increasing the dose of Prevenar was not associated with more injection site reactions and no severe reactions were observed. 84% of the participants reported only mild pain after vaccination{{130 Jackson,L.A. 2007;131 Jackson,L.A. 2005; }}.

The primary disadvantage of the current Prevenar vaccine is that it covers only 7 pneumococcal serotypes and therefore approximately 45% of the pneumococcal infections in adults in Denmark in contrast to Pneumo Novum; which covers approximately 70% {{141 Nielsen,S.V. 1996; 80 Lockhart,S.P. 2006; }}. However a booster effect in HIV-patients has been seen when a conjugated vaccine is administered and followed by the polysaccharide vaccine. This was first observed by Kroon et al{{36 Kroon,F.P. 2000; }} and long term immunity from a conjugated vaccine booster approach was presented by a French group at the 14^th^ Conference on Retroviruses and Opportunistic Infections (CROI) in February 2007{{85 Lévy, Y. 2007; }}. Their primary endpoint was the proportion of vaccine highresponders (defined as 2-fold increase and IgG levels ≥1 µg/mL to at least 5 of 7 pneumococcal serotypes) in the two groups. Although not statistical significant there were more high responders (13% versus 4%, p=0.08) in the prime-boost group versus the polysaccharide vaccine alone group at week 96. The advantage of using a conjugate+polysaccharide pneumococcal vaccination regimen is that the conjugate vaccine primes the immune system resulting in a more immunogenic response to the following polysaccharide vaccine than if the polysaccharide vaccine is given alone. However, 13% high responders after 96 weeks using an optimized pneumococcal vaccination strategy for HIV infected adults is not optimal and there is a need for a better pneumococcal vaccine regimen.

A series of 2 pneumococcal conjugate vaccines (Prevenar) and 1 polysaccharide vaccine (i.e. Pneumo Novum) was found to be immunogenic and safe in American HIV-infected children from 2 to <19 years of age who had low to moderate viral loads and were receiving HAART{{5 Abzug,M.J. 2006; }}. The vaccination approach used in the trial by Abzug et al. was identical to the vaccine regimen planned in this protocol. Since only adults will be included in our study a double dose of Prevenar is planned.

Vaccine adjuvants augment antigen-specific immune responses by physical localization and improved presentation of antigen, and by provocation of inflammatory or innate immune responses{{81 Petrovsky,N. 2004; }}. A key feature in the innate immune system is its capability to detect foreign organisms using a set of cell receptors termed *pattern-recognition receptors* (PRR). The best understood family of PRRs is Toll-Like Receptors. During the past 6-7 years there has been an increasing interest in Toll-Like Receptor 9 (TLR9). TLR9 detects CpG motifs, which are unmethylated CpG dinucleotides within the context of certain flanking bases. CpG motifs are relative common in the genomes of most bacteria and DNA viruses but rare in mammalian genomes. Synthetic oligodeoxynucleotides containing immunostimulatory CpG motifs (CpG ODN) can be used to activate the innate immune system, and subsequently adaptive immunity. The cellular patterns of TLR expression can vary between different species,; for example, TLR9 is found only in plasmacytoid dendritic cells and B cells of humans and other primates while mice also express TLR9 in monocytes and myeloid dendritic cells. While these differences could make it difficult to extrapolate results with CpG ODN in mice to humans, results using CpG ODN as a vaccine adjuvant are actually quite concordant between mice, non-human primates and humans {{40 Krieg,A.M. 2006; }}.

TLR9-stimulation by CpG ODN induces a potent T-helper type I-directed response when administered with almost all types of antigens. CPG 7909, a B-Class CpG ODN of sequence 5’-TCGTCGTTTTGTCGTTTTGTCGTT-3’ is synthesized with a wholly phosphorothioate backbone (Coley Pharmaceutical Group, Wellesley, Massachusetts, USA). This ODN contains three copies of the CpG motif GTCGTT that is optimized for stimulation of the human TLR9 receptor{{41 Cooper,C.L. 2005; }}, however it does also work well in mice.

CPG 7909 has been tested as an adjuvant in several vaccine trials including hepatitis B, influenza, malaria and anthrax. Cooper et al.{{41 Cooper,C.L. 2005; }} used CPG 7909 as an adjuvant to a hepatitis B vaccination schedule in HIV patients and found seroprotective titres in 100% of subjects in the CpG group compared to 63% in the control group after 12 months (p=0.008), and proportion of seroprotected individuals has remained significantly higher in the CPG group even after 5 years (Cooper, personal communication). In a recent study immunotherapy with a ragweed Toll-Like Receptor 9 agonist vaccine for allergic rhinitis appeared to offer long-term clinical efficacy in the treatment of ragweed allergic rhinitis{{66 Creticos,P.S. 2006; }}.

Some of the shortcomings of regular vaccination are:

- Need for several boosts to achieve protection
- Delay in rise of protective antibodies
- Prevalence of vaccine non-responders (as outlined above - this is particularly a problem for immune-compromised individuals)
- Cost of antigen and vaccine production which is a very significant limitation in the development of new conjugated pneumococcal vaccines{{2 Klugman,K.P. 2007; 80 Lockhart,S.P. 2006; }}
- Poorly protective antibodies with low affinity – this has been observed in a number of trials with pneumococcal vaccines in HIV-infected individuals{{13 Feikin,D.R. 2004;30 Rodriguez-Barradas,M.C. 2003; 33 Abadi,J. 1998; }}.
- Falling antibody titres over time.

These shortcomings can potentially be overcome using TLR9-agonists which may:

- Reduce the number of vaccinations required to achieve seroprotection (this was demonstrated in the Engerix and CpG7909 trial{{41 Cooper,C.L. 2005; }})
- Accelerate seroconversion, possibly permitting post-exposure vaccination
- Reduce the proportion of non-responders
- Reduce the amount of antigen required
- Increase antibody avidity and protective activity
- Lead to more sustained antibody levels

(adapted from Krieg 2006{{40 Krieg,A.M. 2006; }})

In a randomized, double-blind controlled Phase I/II dose-escalation study, healthy individuals received three intramuscular injections (using the FDA-approved vaccination regimen of 0, 4 and 24 weeks) of an alum-absorbed HBV vaccine either in saline or mixed with CPG 7909, at doses of 0.125, 0.5 or 1.0 ml{{106 Cooper,C.L. 2004; }}. Hepatitis B surface antigen (HBsAg)-specific antibody responses (anti-HBs) appeared earlier and had higher titres at all time points from 2 weeks after the initial prime up to 48 weeks in CPG 7909 recipients compared with those individuals who received vaccine alone. Moreover, most of the subjects who received CPG 7909 as adjuvant developed protective levels of anti-HBs IgG within just 2 weeks of the priming vaccine dose, compared with none of the subjects receiving the commercial vaccine alone{{41 Cooper,C.L. 2005; }}. In this study, the addition of the TLR9 agonist also increased the proportion of antigen-specific high-avidity antibodies, suggesting enhancement of the late-affinity maturation process in the activated B cells{{82 Siegrist,C.A. 2004; }}.

In the Engerix and CPG 7909 trial in HIV+ subjects, double doses of Engerix were used (bilateral injection of regular dose) together with CPG 7909 {{41 Cooper,C.L. 2005; }}. All subjects in the experimental group received a total of 1 mg CPG 7909 at each immunization (0.5 mg per site). Immunizations were given according to an accelerated schedule of 0, 1 and 2 months rather than the 0, 1 and 6 months used in the healthy volunteer study.

In a trial of non-small-cell lung cancer chemotherapy 112 patients were randomised to receive 0.2 mg/kg CpG7909 by subcutaneous injection in 8-12 cycles with 1-2 weeks interval{{95 Manegold, C. 2005; }}. As in other trials with TLR9-agonists, most common side effects were mild to moderate injection site reactions and transient flu-like symptoms. Grade 3 or 4 neutropenia was more common in the combination arm, which is thought to reflect neutrophil redistribution, but febrile neutropenia and grade 3/4 infections were actually slightly less common in the combination arm than in the chemotherapy alone arm. Thrombocytopenia, a previously recognized phosphorothioate backbone effect that has occurred in all trials of antisense ODN, was seen more commonly in the combination arm, but there was no increase in bleeding events.

The safety profile of several TLR9 agonists in humans has been observed in the clinical trials described above over a more than 1,000-fold dose range from 0.0025–0.81 mg per kg. A maximal tolerated dose in humans has not been reported to date. The primary adverse events are dose-dependent local injection reactions (such as erythema, pain, swelling, induration, pruritus or warmth at the site of injection) or systemic flu-like reactions (such as headache, rigors, myalgia, pyrexia, nausea and vomiting), and are consistent with the known TLR9 agonist mechanism of action. Depending on the dose, systemic symptoms typically develop within 12–24 hours of dosing and persist for 1–2 days. At the low doses used in vaccine trials there seems to be a slight increase in the frequency of injection-site reactions, which are generally mild, above the frequency observed with the vaccine alone. The clinical experience to date indicates that CpG ODN treatment of normal humans, cancer patients or individuals infected with HIV or HCV does not readily induce autoimmune disease.

The immune response to conjugated vaccines with or without CpG ODN has been tested in studies on mice. Chu et al. used conjugated pneumococcal antigen from the Prevenar vaccine and found a 23-fold increase in total antibody response when a CpG ODN was administered with the vaccine compared to the conjugated vaccine alone{{142 Chu,R.S. 2000; }}. Studying the adjuvant effect of CpG ODN with conjugated Haemophilus influenzae type b (Hib) vaccine (using the same carrier as in Prevenar(CRM)) von Hunolstein et al. found a superior immune response when a CpG was added to the vaccine{{148 von Hunolstein,C. 2001; }}. In a study of CpG and pneumococcal vaccine Kovarik et al. found no additive effect of CpG to plain polysaccharide antigen (as used in the Pneumo Novum vaccine){{147 Kovarik,J. 2001; }}. They found an age and serotype-dependent effect of adding CpG to conjugate pneumococcal antigens (using a tetanus toxoid as carrier). The increase in serotype-specific antibodies was only observed in adult mice and was largely dependent on CD4+ cells. Similar results were observed in another study where adding CpG to a conjugated pneumococcal vaccine restored aged mice’s immune response levels to that of young adult mice{{143 Sen,G. 2006; }}. This finding was also linked to CpG ODNs ability to augment CD4+ T-cell help. As depletion of CD4+ T-cells is a key component in the progression of HIV, we hypothesize that the addition of a CpG ODN to a pneumococcal vaccine would be particularly useful in HIV-infected patients.

The optimal time in the course of the disease for immunization of HIV infected individuals is debated. The general opinion is that the sooner - the better, although it’s recognized that patients with low CD4 count (i.e. less than 200) benefit little from vaccination. In this study we have decided to include HAART naïve (individuals who not yet begun antiretroviral treatment) as well as HAART experienced individuals to study and compare vaccination effects in both groups. In relation to CPG 7909 this raises a possible safety issue.

TLR9 activation with bacterial DNA can induce the HIV transcriptional regulatory elements in long terminal repeats (LTRs){{152 Equils,O. 2003; }}, increasing viral replication. HIV-infected humans treated systemically with a B-class ODN that contained a CpG motif showed dose-dependent increases in plasma HIV branched DNA levels, which represent the level of virus in the blood{{153 Agrawal,S. 2003; }}. However, 6 days after the last CpG-infusion plasma HIV had returned to placebo-treated individual’s levels and the magnitude of viremia was dose dependent (no effect on viral replication was observed for low doses CpG). It is important to note that in this study, the CpG ODN was administered in high doses (the lowest dose was 3.2 mg/kg/day, and the highest dose was 4.4 mg/kg/day by continuous intravenous infusion for 8 days, whereas when used as a vaccine adjuvant it is administered IM at a dose of approximately 0.01 mg/kg Furthermore, despite their capacity to induce HIV transcription, CpG ODN can also show anti-HIV activity: the high level of IFN-α- production induced by A-class ODN suppresses HIV replication in human foetal thymus cells{{154 Gurney,K.B. 2004; }}, and B-class ODN can also suppress HIV replication in cultured human cells, albeit in a sequence-independent fashion{{155 Schlaepfer,E. 2004; }}.

Transient increases in HIV viral load have been observed in relation to a variety of immune stimulating events such as flu-vaccination{{160 O'Brien,W.A. 1995; }}, tetanus-vaccination{{163 Stanley,S.K. 1996; }}, oral-cholera-vaccination{{161 Ortigao-de-Sampaio,M.B. 1998; }} and during acute infectious illnesses{{162 Sulkowski,M.S. 1998; }}. IL-2 treatment in humans induces transient increases in HIV DNA but does not promote virological failure or emergence of resistance{{156 Delaugerre,C. 2003; }}. Several recent reviews have concluded that viral load blips (small transient increases in viral load) are not typically associated with the development of resistance mutations and more importantly are not associated with virological or clinical failure of previously adequate HAART{{158 Lee,P.K. 2006;157 Di Mascio,M. 2003; 159 Nettles,R.E. 2005; }}. Furthermore acute infectious illnesses and vaccination has been shown not to induce virological failure or emergence of resistance{{157 Di Mascio,M. 2003;159 Nettles,R.E. 2005; }}. In the Engerix B (+ CPG 7909) trial with 38 HIV infected individuals, no changes in HIV RNA were observed in the CPG 7909 group versus the control group{{41 Cooper,C.L. 2005; }}.Thus, we consider it unlikely that a small intramuscular dose of CPG 7909 should induce virological failure, emergence of resistance or persistent viremia.

CPG 7909 is currently being evaluated as adjuvant to a novel malaria vaccine candidate and is also being tested in a number of cancer treatment trials{{96 US NIH 2007; }}. Searching the US National Institutes of Health (NHI) website of registered clinical trials (www.clinicaltrials.gov) revealed a total of 31 human clinical trials using CPG 7909 and CPG 10101. Ten of the studies were ongoing trials and the rest completed or no longer recruiting patients. More than 2000 subjects were included in the trials receiving one or multiple doses CpG, and subcutaneous injections of up 40 mg CPG 7909 were administered.

A different CpG ODN manufactured by Dynavax was registered in another 5 trials of which the largest – a phase III trial of a novel CpG adjuvanted hepatitis B vaccine (HEPLISAV™) – had an expected total enrolment of 1740 individuals. In all the studies of TLR9-agonists the maximal tolerated dose in humans has never been reached {{40 Krieg,A.M. 2006; }}. Therefore a dosing of 1 mg CPG 7909 with each pneumococcal vaccination appears safe and well below the upper limit used in other TLR9-agonist trials.

Recent trials (TEPVAC and FLUVAC both substudies in the ESPRIT trial{{151 Pett,S.L. 2006;149 The ESPRIT Research Group 2007; 150 Einhaupl,K. 2007; }}) used IL-2 as a vaccine adjuvant. Compared to IL-2, CPG 7909 causes less local and systemic toxicity. Further, an adjuvant effect is seen with only one injection of CPG 7909, whereas series of injections were used in the IL-2 studies. Thus, CPG 7909 appears better suited as an adjuvant than IL-2.

For more extensive information regarding TLR9-agonists and in particular CPG 7909 please refer to Investigator’s Brochure (APPENDIX 1).

Pneumococcal disease is a major source of morbidity and mortality in HIV-patients. HIV-patients are vaccine hyporesponders. A good immune response to pneumococcal vaccination enhances vaccine effectiveness, thereby preventing the morbidity and mortality caused by pneumococcal disease. Even when a prime-boost regimen containing both conjugated and polysaccharide pneumococcal vaccine is used, only 13% of the immunized HIV patients are high-responders at week 96{{85 Lévy, Y. 2007; }}. Recent data indicate that TLR9-agonists have excellent vaccine adjuvant potential and are safe to use in immunocompetent as well as immunocompromised individuals{{40 Krieg,A.M. 2006; }}. The objective of this study is to evaluate the qualitative and quantitave immune response to pneumococcal vaccination with or without CpG7909 in HIV-infected adults. Further, the study will compare the immune response to pneumococcal vaccination in HIV patients receiving HAART with that in patients who have not yet initiated HAART**.**

PROTOCOL

**TITLE: Immune Response to** Toll-like receptor 9-agonist adjuvanted **Pneumococcal Vaccination in HIV Infected Adults**

**PROJECT PHASE:** I

**INDICATION**: HIV infected adults

##### OBJECTIVES:

**Primary Objective**:

- To compare numbers of vaccine high responders - defined as 2-fold increase and IgG levels ≥1 µg/mL to at least 5 of 7 pneumococcal serotypes (by quantitative IgG measurements) – in the CPG 7909 group vs. the control group.

**Secondary Objectives:**

- To compare the qualititative (functional) antibody response to pneumococcal vaccination with or without CPG 7909
- To evaluate safety and tolerance of CPG 7909 as a pneumococcal vaccine adjuvant
- To analyze changes in pneumococcal carrier status after pneumococcal vaccination
- To analyze the predictive value of immune activation/status markers such as CD4 count and sCD163 (at the time of vaccination) for the immune response after pneumococcal vaccination.
- To compare antibody responses to pneumococcal vaccination in patients receiving HAART and patients not receiving HAART
- To analyze cellular activity and response before and after vaccination

##### MAIN ASSESSMENT PARAMETERS:

Efficacy: *Primary*

- Quantitative measurement of specific anticapsular antibodies (7 serotypes)

*Secondary*

- Functional activity of specific anticapsular antibodies (pneumococcal serotypes 6B, 14, 19F and 23F)
- Microbiological changes in pneumococcal pharangyal colonization
- Baseline CD4-count and measurement of sCD163
- Measurements of cellular response, i.e. IFN-γ

Safety/Tolerability:

- Adverse events (AEs).
- Serious adverse events (SAEs)
- Number and intensity of adverse and serious adverse events
- Laboratory tests (hematology, clinical chemistry i.e. viral load (HIV RNA) and CD4-count).
- Physical examination.

**STUDY DESIGN:** Placebo-controlled, randomized, double-blinded study.

**PLANNED TOTAL SAMPLE SIZE:** 94 participants (47 per group).

**CENTER & RECRUITMENT:** The study will be conducted in the Department of Infectious Diseases at Skejby Hospital, Aarhus University Hospital. HIV patients receiving care at other hospitals may also be invited to participate in the study at Skejby Hospital. HIV-infected individuals will be recruited by invitation and patient information sent by regular mail to their home addresses; unless they have previously asked not to receive letters from the department. Study information will also be available in relation to their regular, scheduled consultation at the hospital’s HIV clinic. Individuals interested in study participation may contact the primary investigator or study nurse by phone. A booking for the first visit in relation the study will be made during this conversation and the participant can ask questions regarding the study. After completion of the first study the participant will book a time for his/hers 2^nd^ visit and so fort. A thorough written and oral patient study information will be given before consent is obtained. If the individuals wish to have more time to consider his/hers study participation or if they prefer to have a friend or relative at their side to assist them in their decision they may postpone their decision. Consenting patients that pass the inclusion/exclusion criteria will be enrolled in the study.

400 HIV infected individuals have regular control visits at the Department of Infectious Diseases, Skejby Hospital. Enrolment will be terminated when 94 subjects have been enrolled.

**TEST DRUGS AND FORMULATIONS:** CPG 7909 (a synthetic Toll-like receptor 9-agonist) formulated in PBS buffer.

**TEST DRUG DOSAGE:** 1 mg CPG 7909 (100 μl) mixed with each pneumococcal vaccination.

**CONTROLS:** 100 µl of a neutral PBS buffer (identical in colour and viscocity to the test drug) with each pneumococcal vaccine.

**TEST DRUG AND PLACEBO PREPARATION**

Vial fills of 1.0ml (10mg/ml) CPG 7909 will be shipped from Coley Pharmaceutical Group. The Hospital Pharmacy at Århus University Hospital will import, prepare and label CPG 7909 and placebo as described below. The primary investigator will advise the Hospital Pharmacy of the required amount of doses needed by faxing a complete list of study participants (by study IDs) one day before the participants are scheduled to come. The Hospital Pharmacy is responsible for the safe and appropriate storage of CPG 7909 and placebo until the doses are dispatched to the sponsor/investigator. Upon receipt of the syringes at The Department of Infectious Diseases the sponsor/investigator takes full responsibility for the safe and appropriate storage of the preparations. The Hospital Pharmacy guarantees drug sterility for 24 hours after dispatch.

TREATMENT AND STORAGE OF VACCINES, TEST DRUG AND PLACEBO

Vaccines will be kept in a refrigerator at 5° Celsius in a clearly marked rack at the Department of Infectious Diseases. Test drugs and placebo will be kept on a separate rack in the same refrigerator in identical 1 ml syringes.

**OTHER TREATMENT:** No additional medication or treatment is planned and no changes in regular medication will be made due to the participant’s inclusion in this study. Any changes in medication during the time of the study will not disqualify the individual for further participation in the study and study participation will not interfere with the HIV physician’s decision regarding HAART and other medical treatment received or initiated during the time of the study.

**ROUTE OF ADMINISTRATION:** Intramuscular injection

**BLINDING:** Double-blinded study.

##### ENROLMENT: Randomization;

Eligible patients will be randomized in a ratio of 1:1 to receive **pneumococcal vaccination with or without CPG 7909**. Random allocation lists will be generated by computer by the hospital pharmacy. A complete randomization list will be stored at the hospital pharmacy. Cluster randomization will be used to obtain similar distributions of on-HAART and HAART-naïve subjects in the two treatment arms. Subjects will be randomized in blocks of 6. Sealed envelopes revealing the subjects’ treatment allocation will be made by the Hospital Pharmacy and transferred to a safe at the Department of Infectious Diseases before initiating the study. If indicated (i.e. in the case of a SUSAR (see below)) a subject’s group allocation can be revealed without unmasking the entire study.

Participants will receive their study ID after passing inclusion/exclusion criteria (according to treatment; +HAART/-HAART). Participants will be entered into the study and given study ID according to the chronological order in which they are included (i.e. the first subject in the study will be assigned study ID #1 if HAART naïve, or study ID #43 if on HAART, the 2^nd^ subject will receive the next available study ID according to HAART status and so fort). Registration is done by the investigator by highlighting the next available number on the entry list and by adding randomization labels to the front of the subjects CRF. Participants will be blinded to their assigned vaccine adjuvant (placebo or CPG 7909). Investigators conducting the immunization will also be blinded to the subject’s treatment allocation and to the contents of the test drug/placebo preparation.

**IMMUNIZATION**

Vaccines will be kept in their original container according to manufacturer’s description and mixed with the adjuvant immediately before immunization. The formulation of vaccine plus adjuvant/placebo will be prepared by the physician. Dosing of adjuvant/placebo and sealing of syringes will be done by the Hospital Pharmacy. Adjuvant (CPG 7909 or Placebo) will be delivered from the Hospital Pharmacy in a sealed 1 ml syringe. Each syringe will be labeled with the following information: study ID, Batch number and time+date of dispatch. Batch number and study ID will be recorded in the subject’s CRF. Immunization will be done in the left or right upper deltoid muscle at the preference of the subject.

**ACCESS TO AND STORAGE OF RANDOMIZATION ENVELOPES**

Randomization envelopes will be locked away at the Department of Infectious Diseases, Skejby hospital – Aarhus University Hospital, Brendstrupgaardvej, 8200 Aarhus N. Access to randomization envelopes can be granted through contact to the study investigators or in rare cases (i.e. if primary and co-investigators are unavailable) thru the senior Infectious Disease physician on call.

**DURATION OF TRIAL FOR EACH PARTICIPANT:** 10 months from 1^st^ vaccination to last follow-up.

##### SUBJECT WITHDRAWAL FROM THE STUDY:

From an analysis perspective, a “withdrawal” from the study is any subject who did not come back for the concluding visit foreseen in the protocol.

A subject qualifies for “withdrawal” from the study when no study procedure has occurred, no follow-up has been performed and no further information has been collected for this subject from the date of withdrawal/last contact.

Investigators will make an attempt to contact those subjects who do not return for scheduled visits or follow-up.

Information related to the withdrawal will be documented on the Study Conclusion page on the CRF. The investigator will document whether the decision to withdraw from the study was made by the subject or the investigator and which of the following possible reasons was responsible for the withdrawal:

- Participant’s withdrawal of informed consent
- Protocol violation
- A serious adverse event (SAE)
- Non-serious adverse event
- Lost to follow-up
- Other (specify)

**Withdrawals will not be replaced.**

##### SUBJECT WITHDRAWAL FROM INVESTIGATIONAL PRODUCT

A withdrawal from the investigational product is any subject who does not receive the complete treatment, i.e. when no further planned dose is administered from the date of withdrawal. A subject withdrawal from the investigational product may not necessarily be withdrawn from the study as further study procedures or follow-up may be performed (safety or immunogenicity) if planned in the protocol.

Information related to premature discontinuation of the investigational product will be documented in the CRF. The investigator will document whether the decision to discontinue further vaccination was made by the subject or the investigator and which of the following reasons was responsible for the withdrawal:

- Serious adverse event
- Non-serious adverse event
- Other (specify)

Unblinding of the subject will documented in the CRF.

##### DATA TO BE INCLUDED IN THE CASE REPORT FORM:

- Birthday, sex, race, height, weight, study number
- Adverse events reported by subject including starting point and duration (time to resolution)
- Positive findings during physical examination
- Medical history
- Other vaccinations received outside the study during the study period
- Any changes in regular medication during the time of study
- Pre-existing conditions or signs and/or symptoms present in a subject prior to the start of the study/first vaccination
- All laboratory findings during the time of the study

##### REASONS FOR “BREAKING” A PARTICIPANTS RANDOMIZATION CODE:

- SAE and a strong degree of probability that the event is an adverse reaction to a vaccination received during this trial
- Unexpected death of a study participant

##### SPONSOR’S TERMINATION OF STUDY:

- The sponsor reserves the right to temporarily suspend or prematurely discontinue this study for reasons including, but not limited to, safety or ethical issues or severe recruitment difficulties.

##### PARTICIPANT INCLUSION CRITERIA:

1. Written informed consent and authority statement provided according to local regulatory and ethical practice using a participant information sheet and informed consent form approved by the responsible Ethics Committee.
2. Male or female participants aged >= 18 years.
3. HIV-seropositive individuals

##### PARTICIPANT EXCLUSION CRITERIA:

1. Pregnancy as determined by a positive urine beta-hCG (if female).
2. Participant unwilling to use reliable contraception methods for the duration of the trial. Reliable methods of birth control include: pharmacologic contraceptives including oral, parenteral, and transcutaneous delivery; condoms with spermicide; diaphragm with spermicide; surgical sterilization; vaginal ring; intrauterine device; abstinence; and post-menopause (if female).
3. Currently breast-feeding (if female).
4. Latest CD4 count < 200 x10^6^ cells/µL
5. Viral load (HIV RNA) > 50 copies/mL **if** on HAART (defined as at least three antiretrovirals including either a protease inhibitor or a NNRTI, i.e. combivir 300/150 mg x2 + stocrin 600 mg x1 for a minimum of 6 months)
6. Previous enrollment in this study.
7. Any medical, psychiatric, social, or occupational condition or other responsibility that, in the judgment of the Principal Investigator (PI), would interfere with the evaluation of study objectives (such as severe alcohol abuse, severe drug abuse, dementia).
8. Unable to follow protocol regimen
9. Pneumococcal vaccination 5 years or less prior to inclusion
10. Planned participation in other vaccination trials during the time of the study

##### PROCEDURES:

Consenting participants that pass the inclusion/exclusion criteria will be enrolled in the study. Blood samples for baseline parameter measurements will be drawn before proceeding to immunization.

At randomization, participants will be allocated 1:1 one of two study regimens:

- **Experimental group**: Double paediatric dose of 7-valent conjugate pneumococcal vaccination (Prevenar®, Wyeth) + 1 mg CPG 7909 (day 0), double paediatric dose of 7-valent conjugate pneumococcal vaccination (Prevenar®, Wyeth) + 1 mg CPG 7909 (day 90) and single dose of 23-valent polysaccharide vaccine (Pneumo Novum®, Sanofi Pasteur MSD) + 1 mg CPG 7909 (day 270)
- **Control group**: Double paediatric dose of 7-valent conjugate pneumococcal vaccination (Prevenar®, Wyeth) + 100 μl of placebo (day 0), double pediatric dose of 7-valent conjugate pneumococcal vaccination (Prevenar®, Wyeth) + 100 μl of placebo (day 90) and single dose of 23-valent polysaccharide vaccine (Pneumo Novum®, Sanofi Pasteur MSD) + 100 μl of placebo (day 270).

Blood samples are drawn and follow-up by the physician includes physical examination and medical history, registration of AEs/SAEs, vaccination history outside the study and any other information that may be relevant to document in the CRF. A concluding visit is scheduled at day 300.

A subject who returns for the concluding visit or is available for the concluding contact foreseen in this protocol is considered to have completed the study.

##### VACCINES AND TEST DRUG/PLACEBO INJECTIONS:

All subjects are dosed at 0, 90 and 270 days. All immunizations are done in the deltoid muscle of the right or left arm (according to the participants preference).

- At day 0 and 90 study participants receive one intramuscular injection of double the recommended paediatric dose of Prevenar 1.0 ml + 0.1 ml test drug (CPG 7909)/placebo. In both cases, the volume injected into the arm is 1.1 ml.
- At day 270 study participants receives one intramuscular injection of 0.5 ml Pneumo Novum + 0.1 ml test drug(CPG 7909)/placebo. In all cases, the volume injected into the arm is 1.1 ml.

Investigators and participants are not aware of whether experimental or control injection is administered. The volume and appearance of each injection product are identical. The exact product number of each test drug/placebo and batch number prepared by the hospital pharmacy will be recorded in the individuals CRF as well as the volume of any remaining fluid in the syringe .

Immunization and follow-up is conducted by investigators and study nurses at the Department of Infectious Diseases at Skejby Hospital, Aarhus University Hospital.

**TEST DRUG(CPG 7909)/PLACEBO AND VACCINE SUPPLIES**

The Hospital Pharmacy Aarhus Hospital, Aarhus University Hospital will supply the following study drug and placebo sufficient number of doses to administer to all subjects as described in the present protocol:

- 141 doses of 0.1 ml CPG 7909 formulated in PBS buffer
- 141 doses of 0.1 ml placebo

At least an additional 10% of their respective amounts will be supplied for replacement in case of breakage, bad storage conditions or any other reason that would make the test drug/placebo or vaccine unusable (i.e. given by mistake to another subject).

Vaccines will be delivered directly to the Department of Infectious Diseases and stored according to manufacturer’s prescriptions.

- 374 doses of 0.5 ml Prevenar, Wyeth
- 94 doses of 0.5 ml Pneumo Novum, Sanofi Pasteur

**TEST DRUG/PLACEBO ACCOUNTABILITY**

At all times the figures on supplied, used and remaining test drug/placebo doses should match. At the end of the study, it must be possible to reconcile delivery records with those of used and unused stocks. An explanation must be given of any discrepancies.

**EVALUATIONS:**

Study assessment will be done by investigators and include: Written informed consent, inclusion/exclusion criteria, medical history, demographics, pregnancy test (Women of childbearing potential only), physical and overall assessment of study eligibility.

##### PRIMARY EFFICACY PARAMETER AND ANALYSIS OF ANTIBODY RESPONSE

The study is powered to detect differences between the experimental group and the control group in Pneumococcal vaccine high responders defined as 2-fold increase and IgG levels ≥1 µg/mL to at least 5 of 7 pneumococcal serotypes (by quantitative IgG measurements). The study is not powered to detect differences in the incidence of pneumonia or confirmed pneumococcal disease invasive/non-invasive. This would require a substantial number of participants and a longer follow-up period. The most widely used measurement of immune response to pneumococcal vaccination is quantitative detection of serotype specific anticapsular antibodies. Recent data indicate that the specificity of this method can be improved by incorporation a 22Fabsorption step; thereby removing crossreacting antibodies of low avidity{{13 Feikin,D.R. 2004; }}. Quantitative measurements are done at Statens Seruminstitute, Copenhagen.

##### SECONDARY EFFICACY PARAMETER AND ANALYSIS OF ANTIBODY RESPONSE

Measuring the quantitative amount of serotype specific anticapsular antibodies does not give any information the functionality of the antibodies. This can be measured by a flow-cytometric opsonophagocytic assay and gives indirect information on the antibodies ability to opsonize and facilitate killing of invading pneumococci{{75 Martinez,J.E. 2006; }}. The result of a recent WHO seminar held in January 2007 on standardization of opsonophagocytic assays is expected to be published soon and any possible changes to the method described below will be incorporated.

*Qualitative* analysis are done using a flowcytometric opsonophagocytic assay which measures functional (opsonophagocytic) activity (OPA) of the serotype specific antibodies. In short: Eight twofold dilutions are made in OPA buffer from 10 μl of test serum. A 20-μl aliquot of either multiplex bacteria or multiplex bead suspension containing 1x10^5^ of each of the target pneumococcal serotype or pneumococcal polysaccharide-conjugated beads is added to each

well, and the plate is incubated for one hour at 37°C with horizontal shaking (200 rpm). Following this, 20 μl of sterile serum from 3- to 4-week-old baby rabbit serum (Pel-Freez, Brown Deer, Wis.) is added to each well except for HL60 cell control wells, which receives 20 μl of OPA buffer. After incubation at 37°C for 20 min with shaking (200 rpm on an orbital shaker), 30 μl of washed HL60 polymorphonuclear leukocytes (PMNs) (2.5 μ 104/ml) are added to each well, resulting in an effector-to-target ratio of 1:4 (for each target type). The final well volume is 80 μl, with the first well of a dilution series containing a 1:8 final dilution. The plate is then incubated for 60 min with shaking at 37°C. An additional 80 μl of OPA buffer is added to every well to provide sufficient volume for flow cytometric analysis and the well contents transferred to microtiter tubes (Bio-Rad, Hercules, Calif.). Up to 12 serum samples can be assayed per plate, including a quality control sample. Flow analysis are done by Flow Applications, Inc, Ill, USA{{75 Martinez,J.E. 2006; }}.

##### PNEUMOCOCCAL CARRIAGE

Pneumococcal vaccination can affect pharyngeal carriage of pneumococci. Pneumococcal pharyngeal colonization may also affect the immune response to pneumococcal vaccination{{86 Frazao,N. 2005; }}. Therefore it is important to establish carrier status before and after pneumococcal vaccination. Oropharyngeal colonization will be tested in the posterior pharynx using a BBL culture swap (Becton Dickson Microbiology Systems, Cockeysville, MD, USA) thru the oral cavity. Samples will be labeled with the individuals study ID number, frozen at -20°C within few hours and later shipped to Statens Serum Institut, where isolation, culturing and serotyping will take place. This will take place at day 0 and again during follow-up at day 270.

##### SAFETY/TOLERABILITY ASSESSMENT PARAMETERS:

The investigator is responsible for the detection and documentation of events meeting the criteria and definition an adverse event (AE) or serious adverse event (SAE) as provided in the protocol. During the study, when there is a safety evaluation, the investigator or site staff will be responsible for detecting AEs and SAEs, as detailed in this section of the protocol.

Each subject will be instructed to contact the investigator immediately should the subject manifest any signs or symptoms they perceive as serious.

##### ADVERSE EVENTS (AEs):

An AE is any untoward medical occurrence in a clinical investigation subject, temporally associated with the use of a medicinal product, whether or not considered related to the medicinal product.

An AE can therefore be any unfavorable and unintended sign (including an abnormal laboratory finding), symptom or disease (new or exacerbated) temporally associated with the use of a medicinal product.

In this study an AE will be graded according to the Common Toxicity Criteria, version 2.0. The criteria are located in the Appendix 2.

##### SERIOUS ADVERSE EVENT (SAE) DEFINITION:

An adverse event occurring during a clinical trial is any undesirable experience associated with the use of a medical product in a participant. The event is serious and will be reported to the regulatory authority when the participant outcome is:

1. Death
2. Life-Threatening
3. Hospitalization (initial or prolonged)
4. Disability
5. Requiring Intervention to Prevent Permanent Impairment or Damage
6. Congenital disorder/anomaly (for pregnant women)

In this study we will follow a cohort of 94 HIV-infected individuals for 10 months each giving a total observation time of approximately 78 person years. As noted in the background section of this protocol HIV-infected individuals have a much higher morbidity and mortality than an aged-matched background population. Thus, we can expect to have anywhere between 10 and 20 SAEs during the time of the study. All of these SAEs will be investigated to determine if the event fulfills criteria for being a SUSAR.

##### SUSPECTED UNEXPECTED SERIOUS ADVERSE EVENT REACTION (SUSAR) DEFINITION:

A Suspected Unexpected Serious Adverse Reaction (SUSAR) occurring during the study and is vil be reported to the Danish Medicines Agency on a **Council for International Organizations of Medical Sciences** (CIOMS) form (Appendix 3):

- The event must be a SAE.
- There must be a certain degree of probability that the event is an adverse reaction on the administered drug.

The adverse reaction must be unexpected, that is to say, not foreseen in the Investigator’s Brochure (for an unauthorised medicinal product) or in the summary of product characteristics (for Prevenar and Pneumo Novum).

##### SUBMITTING SUSPECTED UNEXPECTED SERIOUS ADVERSE EVENT REACTION (SUSAR) REPORTS

The investigator will report a SAE/SUSAR to the sponsor within **24 hours of his/her becoming aware of the event.** SUSARs will be reported to the Danish Medicines once the sponsor determines that the event meets protocol definitions of a SUSAR. The sponsor will fax the SUSAR report/CIOMS form to the Danish Medicines Agency **within 7/15 DAYS of his becoming aware of the event.** The investigator will provide an assessment of causality at the time of the initial report. In the event of a death determined by the investigator to be related to vaccination/test drug a fax must be sent to the study monitor.

CLINICAL LABORATORY PARAMETERS AND OTHER ABNORMAL ASSESSMENTS QUALIFYING AS ADVERSE AND SERIOUS ADVERSE EVENTS

Abnormal laboratory findings or other abnormal assessments (i.e. by physical examination) that are judged by the investigator to be clinically significant will be recorded as AEs or SAEs if they meet the criteria outlined above. Clinically significant abnormal laboratory findings or other abnormal assessments that are detected during the study or are present at baseline and significantly worsen following the start of the study will be reported as AEs or SAEs.

The investigator will exercise his or her medical and scientific judgement in deciding whether an abnormal laboratory finding or other abnormal assessment is clinically significant.

##### FOLLOW-UP OF ADVERSE EVENTS AND SERIOUS ADVERSE EVENTS AND ASSESSMENT OF OUTCOME

After the initial AE/SAE report, the investigator is required to proactively follow each subject and provide further information to the study group on the participant’s condition.

All AE/SAEs documented at a previous visit/contact and designated as not recovered/not resolved or recovering/resolving will be reviewed at subsequent contacts/visits.

Investigators will follow-up subjects:

- With SAEs or subjects withdrawn from the study as a result of an AE, until the event has resolved, subsided, stabilized, disappeared, the event is otherwise explained, or the subject is lost to follow-up;
- Or, in the case of other non-serious AEs, until they complete the study or are lost to follow-up

Clinically significant laboratory abnormalities will be followed up until they have returned to normal, or a satisfactory explanation has been provided. Additional information (including, but not limited to, laboratory results) relative to the subsequent course of such an abnormality noted for any subject must be made available to the Study Monitor.

Outcome of any non-serious adverse event occurring within 30 days post-vaccination, any specific AEs (new onset of chronic illness, rash, events prompting emergency room visit and/or physician office visit for non-routine care and any event related to vaccine efficacy (i.e. pneumococcal disease) or any SAE reported during the entire study will be assessed as:

- Recovered/resolved
- Not recovered/not resolved
- Recovering/resolving
- Recovered/resolved with sequelae
- Fatal (SAEs only)

##### TREATMENT OF ADVERSE EVENTS

Treatment of any adverse event is at the sole discretion of the investigator and according to good medical practice. Any medication administered for the treatment of an AE should be recorded in the subjects CRF.

##### SAFETY UPDATES

A periodic safety update report (PSUR) is a report describing the safety of a medicinal product. The primary investigator will submit a PSUR once annually to the Danish Medicines Agency and the regional ethical comiteé until the trial is completed. Among other things, such a report will include a list of the adverse reactions known to the sponsor, an estimate of the number of humans treated with the medicinal product (CPG 7909), an overall assessment of the medicinal product’s safety profile suggestions for new initiatives as regards safety, if relevant

***After the trial has ended a complete safety and study report will be submitted to the Danish Medicines Agency and Coley Pharmaceutical Group, Inc.***

DMA

**Reporting Serious Adverse Events
during the *ITAP study***

Sponsor

Investigator

All investigators

Coley P.G.

DMA.

Reg. Ethical Committee

SAEs according to definitions

in the study protocol

List of all SUSARs every 6 months

According to the pharmaco-

vigilante agreement provided By Coley Pharmaceutical Grp

List of all SAEs once annually + report on the safety of study participants

All SUSARs reported within 7/15 days using a CIOMS form

List of all SAEs once annually + report on the safety of study participants

*SAE: Serious Adverse Event*

*SAR: Serious Adverse Reaction*

*SUSAR: Suspected Unexpected Adverse Reaction*

*DMA: Danish Medicines Agency*

##### SOLICITED ADVERSE EVENTS

A four day follow-up (day 1 to 4) of solicited local (injection site) and general adverse events will be performed after study vaccine administration. Data concerning the AEs will be solicited using diary cards provided by the sponsor-investigator and AEs will be listed as detailed on page 25.

**UNSOLICITED ADVERSE EVENTS**

During the entire study period (from day 0 to month 10) follow-up will be conducted for the occurrence of specific AEs which consist of the following:

- New onset of chronic illness(es) (e.g., autoimmune disorders, asthma, type I diabetes)
- Rash (i.e. hives)
- Any conditions prompting any ER/non-routine physician office visits (i.e. office visits not related to well-child care, vaccination, injury or common acute illnesses such as upper respiratory tract infections, otitis media, pharyngitis, gastroenteritis)

##### EVALUATING ADVERSE EVENTS AND SERIOUS ADVERSE EVENTS

##### ASSESSMENT OF INTENSITY

Intensity of AEs will be assessed as outlined in the common toxicity criteria (CTC) version 2.0 (APPENDIX 2).

The investigator will make assessment of the maximum intensity that occurred over the duration of the event in cases where the event was not registered in the participant’s diary (i.e. unsolicited AEs, SAEs, APPENDIX 4). The assessment will be based on the investigator’s clinical judgement.

**ASSESSMENT OF CAUSALITY**

The investigator is obligated to assess the relationship between the investigational product and the occurrence of each AE/SAE. The investigator will use clinical judgement to determine the relationship- Alternative causes, such as natural history of the underlying diseases, concomitant therapy, other risk factors and the temporal relationship of the event to the investigational product will be considered and investigated. The investigator will also consult the Investigator Brochure and vaccine product information in the determination of his/hers assessment.

All solicited local (injection site) reactions will be considered causally related to vaccination. Causality of all other AEs should be assessed by the investigator.

##### DATA EVALUATION: CRITERIA FOR EVALUATION OF OBJECTIVES

All endpoints will be compared between the experimental vaccine group (+CPG 7909) and the control vaccine group (+placebo).

A substudy will compare endpoints in the two (non-randomised) treatment groups (on HAART vs. no HAART)

**PRIMARY ENDPOINTS:**

*At six months after 2^nd^ vaccination with Prevenar.*

- Pneumococcal vaccine high responders defined as 2-fold increase and IgG levels ≥1 µg/mL to at least 5 of 7 pneumococcal serotypes (by quantitative IgG measurements)

**SECONDARY ENDPOINTS:**

**IMMUNOGENICITY**

*At three months after 1^st^ vaccination with Prevenar.*

- Pneumococcal vaccine high responders defined as 2-fold increase and IgG levels ≥1 µg/mL to at least 5 of 7 pneumococcal serotypes (by quantitative IgG measurements)
- Opsonophagocytic activity for serotypes 6B, 14, 19F and 23F expressed as titers
- Serotype-specific antibody response defined as 2-fold increase and IgG levels ≥1 µg/mL
- Serotype-specific antibody response defined as change in IgG levels

*At six months after 2^nd^ vaccination with Prevenar.*

- Opsonophagocytic activity for serotypes 6B, 14, 19F and 23F expressed as titers
- Serotype-specific antibody response defined as 2-fold increase and IgG levels ≥1 µg/mL
- Serotype-specific antibody response defined as change in IgG levels
- Cellular immune response

*At one month after vaccination with Pneumo Novum.*

- Pneumococcal vaccine high responders defined as 2-fold increase and IgG levels ≥1 µg/mL to at least 5 of 7 pneumococcal serotypes (by quantitative IgG measurements)
- Opsonophagocytic activity for serotypes 6B, 14, 19F and 23F expressed as titers
- Serotype-specific antibody response defined as 2-fold increase and IgG levels ≥1 µg/mL
- Serotype-specific antibody response defined as change in IgG levels
- Geometric Mean Antibody Concentrations With the Standard Enzyme Immunoassay for serotypes 1, 4, 7F, 9V, 14, 18C and 19F

**PHARYNGEAL COLONIZATION**

*At six months after 2^nd^ vaccination with Prevenar.*

- Number of individuals with pneumococcal colonization

**PREDICTORS OF ANTIBODY RESPONSE**

*At baseline.*

- Predictors for vaccine response at six months after 2^nd^ vaccination with Prevenar,

**SECONDARY ENDPOINTS:**

**REACTOGENICITY AND SAFETY IN ALL SUBJECTS**

**ANALYSIS POPULATIONS:**

**Safety population:** all patients who received at least one vaccination.

- Occurrence of solicited and general symptoms during the 4-day (day 0 to Day 3) period after each vaccination dose
- Occurrence of unsolicited symptoms up to 1 month after each vaccination
- Changes in CD4-count and viral load during the study

Safety will be assessed by physical examination, adverse events (according to common toxicity criteria version 2.0), laboratory tests, and HIV control parameters (HIV RNA and CD4-count).

**STATISTICAL ANALYSES**

**Baseline characteristics**

Differences between study groups at day 0 will be assessed by Mann-Whitney rank sum test (continuous variables) and Chi-square test (dichotomous and categorical variables).

**Primary endpoint**

Prevalence ratios of high responders at six months after 2^nd^ vaccination with Prevenar, comparing the two vaccination scheme groups (with/without CPG 7909), will be estimated by Poisson regression adjusted by age, CD4 cell count at baseline and HAART (on HAART vs. no HAART) at baseline.

**Secondary endpoints**

Comparison of endpoints between the study groups will be done by Poisson regression (dichotomous endpoints) or linear regression (continuous endpoints), adjusted for appropriate potential confounders.

Predictors for achieving a high vaccination response (classified as a high responder) at six months after 2^nd^ vaccination with Prevenar will be estimated by multivariate Poisson regression.

**Safety data**

Safety data will be listed and summarized. Adverse events will be reported in a table similar to the one below, described as proportions with 95% confidence intervals.


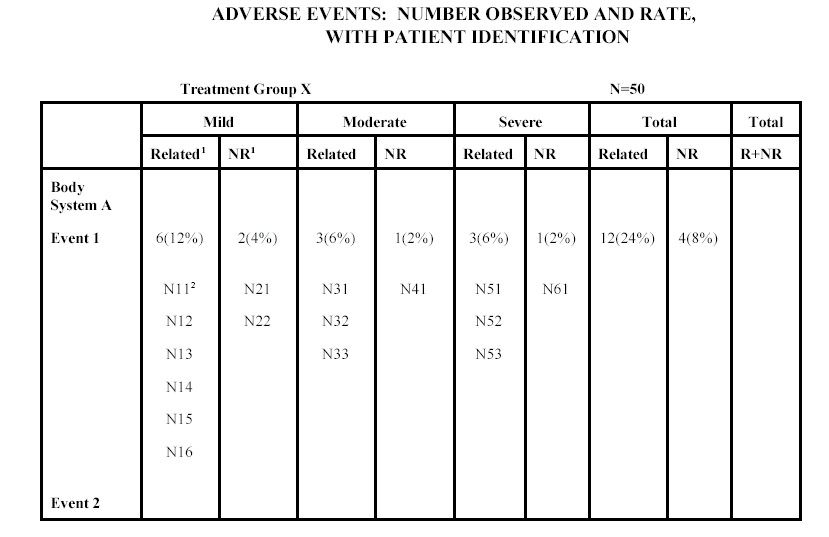


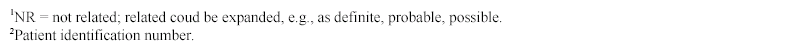


##### ESTIMATED SAMPLE SIZE

**Intention-to-treat (ITT) population:** all randomized participants

Sample size is calculated for the primary endpoint (prevalence ratios of **high responders at six months after 2^nd^ vaccination with Prevenar**, comparing the two vaccination scheme groups). Setting the probabilities of Type I and Type II error to:

- Type I error probability (a) = 0.05 (two-sided).
- Type II error probability (ß) = 0.20 (power = 1 - ß = 0.80).
- Primary endpoint: proportion of vaccine highresponders (defined as 2-fold increase and IgG levels ≥1 µg/mL to at least 5 of 7 pneumococcal serotypes).
- N is the number of participants needed in each group.

| Control\CpG | 0.50 | 0.55 | 0.60 | 0.65 | 0.70 |
| --- | --- | --- | --- | --- | --- |
| 0.20 | 39 | 29 | 23 | 18 | 15 |
| 0.25 | 58 | 41 | 31 | 24 | 19 |
| 0.30 | 93 | 61 | **42** | 31 | 24 |
| 0.35 | 170 | 96 | 62 | 43 | 31 |
| 0.40 | 388 | 173 | 97 | 62 | 42 |

Assuming a prevalence of 30% in control vaccine the group and a prevalence of 60% in the experimental vaccine group a sample size of 42 patients per group is required to detect a difference in prevalence estimated by Poisson regression. The expected drop-out percentage is set to 10%. Thus, a total of 94 subjects are needed in the study.

In accordance with the approach recommended by regulatory authorities, the two-sided 95% confidence interval (CI) of the immune response difference will be calculated.

Should the targeted number of participants not be reached within 12 months from study initiation the primary investigator can decide to stop the study if he considers reaching the expected 94 patients unlikely under the given circumstances. In that case all statistical analysis will then be based on the number of patients included at the time of study termination.

##### ACCESS TO CRFs, PARTICIPANT RECORD AND STUDY RELATED DOCUMENTS

For monitoring, auditing and/or inspection purposes access to CRFs, participant medical records and all study related documents will be granted promptly (by the primary study investigator and/or the scientific advisor) if requested by the study monitor unit, Danish Medicines Agency and/or Regional Ethical comitée.

##### STUDY QUALITY CONTROL AND ASSESSMENT

It is the Sponsor’s responsibility to plan and ensure that the study is conducted according to Good Clinical Practice guidelines, the 1996 version of the Declaration of Helsinki and Danish ethical and legal authority regulations. This implies thorough registering and reporting data according to the CONSORT guidelines{{87 Moher,D. 2001; }}.

It is the Sponsor’s responsibility to establish and maintain a quality control system that guarantees the quality in all aspects of the study.

##### ETHICAL CONSIDERATIONS

Applications to the Danish Data Protection Agency, Regional Ethical committee and Danish Medicines Agency will be submitted. The 23-valent polysaccharide pneumococcal vaccine (i.e. Pneumo Novum) has been on the market since early 1980’s and has been very well tolerated in both immunocompetent and immunocompromised individuals. Only some minor adverse effects (local swelling, nausea, local erythema) and very few serious adverse have been reported even in severely immune compromised patients. The 7-valent conjugated pneumococcal vaccine (Prevenar) has been part of the American children vaccination program since 2000 and several Western European countries have since followed (in accordance with WHO recommendations). Post-marketing surveillance and vaccine trials (including trials on both HIV-positive children and adults) have shown that the vaccine is very well tolerated{{21 Madhi,S.A. 2005;20 Madhi,S.A. 2005; 80 Lockhart,S.P. 2006; 22 Klugman,K.P. 2003; 3 Kamchaisatian,W. 2006; 15 Feikin,D.R. 2001; }}. We therefore consider it unlikely that the vaccines will cause more than minor temporally discomfort. In the background section of this protocol and in the investigator’s brochure (APPENDIX 1) we have discussed the safety of TLR9-agonists. In general the safety of TLR9-agonists has been very good in all trials with TLR9-agonists. At low doses in vaccine trials (like the 1 mg planned in this study) there seems to be a slight increase in the frequency of injection site reactions relative to vaccine alone, but not increased severity as these are generally mild. The clinical experience to date indicates that CPG 7909 treatment (and TLR9-agonist treatment in general) of normal humans, cancer patients or individuals infected with HIV or Hepatitis C does not induce autoimmune disease, kidney disease, liver disease, spleen or bone marrow disease. In all the studies of TLR9-agonist the maximal tolerated dose in humans has never been reached. Therefore a dosing of 1 mg CPG 7909 with each pneumococcal vaccination appears safe and well below the upper limit used in other TLR9-agonist trials. The benefit for all study participants who receive all study vaccinations is that they receive the most effective pneumococcal vaccination known at present time. Our hypothesis is that TLR9-agonist adjuvanted pneumococcal vaccination will substantially improve the magnitude and duration of seroprotection in HIV-infected individuals; thereby reducing morbidity and mortality caused by pneumococcal disease. Thus, on a individual level participants reduce their risk of pneumococcal infection and on a larger scale they contribute to our knowledge of TLR9-enhanced pneumococcal vaccination. Not only HIV-patients; but also elderly, cancer patients and other immune compromised individuals could benefit from this vaccination approach in the future.

##### WRITTEN CONSENT AND PARTICIPANT INFORMATION

Study participants will be recruited when they come for their regular HIV treatment control at the HIV treating center. Written consent will only be obtained after a thorough patient oral and written information has been given by the investigator. If the individual wishes to have more time to consider his or her study participation, he or she can take the information home and contact the study center when a decision has been made.

##### DATA HANDLING AND MANAGEMENT

Blood samples will not be labelled with information that directly identifies the subject but will be coded with the study identification number for the subject.

Samples collected may only be used for the purposes described in this protocol.

Any sample testing will be done in line with the consent of the individual subject.

CRFs and results from blood samples will not be labelled with information that directly identifies the subject but will be coded with the study identification number for the subject. They will be kept in a locked room where access is restricted to study investigators. The investigators are responsible for the data are handled carefully and according to the Danish Data Protection Agency, Regional Ethical committee and Danish Medicines Agency regulations.

The subject identification code will be kept separate from the above subject related information. Access to the identification code is restricted to the primary study investigator and sponsor.

After completion of data collection, all data and participant samples will be entered into a database and the study identification number for the subject will be entered without further information that could directly identify the subject. The identification list identifying each subject by name and Danish Civil Registry Number will be kept in a safe at another location in the Department of Infectious Diseases, Skejby Hospital. Blood samples will be destroyed after completion of all primary and subanalysis. Study data will be available for inspection 10 years after study completion.

##### FINANCING

The study receives no financial support from any of the involved medical companies (Sanofi Pasteur, Wyeth or Coley Pharmaceutical Group). The principal investigator and co-authors of this protocol have no financial interest in the above mentioned companies and receive no personal funding from them during the study. The main funding for the study is achieved thru private fundings and The Clinical Institute at Aarhus University. The laboratory analysis are paid for by the Department of Infectious Diseases, Skejby Hospital. Statens Serum Institute and Flow Applications, Inc. contribute to study financing by offering discounted prices for their laboratory analysis. A “Clinical Trial Agreement” is signed with Coley Pharmaceutical Group who provides CPG 7909 for the study free of charge.

##### PARTICIPANT INSURANCE

All participants are covered through the Danish national patient insurance, through the law of financial compensation from damage due to medicinal products and can also request compensation for personal injuries or sequela from medical research projects.

##### TRIAL REGISTRATION

The trial will be registered on www.clinicaltrials.gov where details of the study and a synopsis will be made publicly available.

##### PUBLICATION OF STUDY RESULTS

One major international publication with key data on antibody response and safety/tolerability of a prime-boost pneumococcal vaccination regimen with or without TLR9-agonist is planned. A final list of authors is not ready as it depends on participating investigator’s contributions to the study. However the following authors are expected to be major contributors to the study:

- Ole Schmeltz Søgaard, MD, Department of infectious diseases, Skejby Hospital – Aarhus University Hospital
- Lars Østergaard; MD, PhD, DMSc, Medical director, A/Prof., Department of infectious diseases, Skejby Hospital – Aarhus University Hospital
- Henrik Carl Schönheyder; MD, PhD, DMSc, Professor, Department of Clinical Microbiology, Aalborg Hospital – Aarhus University Hospital
- Nicolai Lohse, MD, PhD, Department of Clinical Epidemiology, Aarhus University Hospital
- Arthur M Krieg, [MD](http://www.coleypharma.com/coley/manage_bio_krieg.html), Senior Vice President, Research and Development
  Chief Scientific Officer, Coley Pharmaceutical Group
- Zitta Barella Harboe, MD, Statens Serum Institute, Copenhagen

One or two minor publications on pneumococcal carriage and inflammatory markers as predictors of antibody response are also expected to be published in international medical journals.

A study report in the form of an article will be forwarded to Danish Medicines Agency, The GCP-unit and Coley Pharmaceutical Group after trial completion.

# PROTOCOL SYNOPSIS

*Title:* ****Immune Response to**** Toll-like receptor 9-agonist adjuvanted ****Pneumococcal Vaccination in HIV Infected Adults****

*****Indication/Study population:* Immunization of HIV-infected individuals on HAART using a regimen of two conjugated pneumococcal vaccines (Prevenar) and one polysaccharide pneumococcal vaccine (Pneumo Novum) with or without CPG 7909.****

*Rationale*: Pneumococcal disease is a major source of morbidity and mortality in HIV-patients. HIV-patients are vaccine hyporeponders. A good immune response to pneumococcal vaccination enhances vaccine effectiveness, thereby preventing the morbidity and mortality caused by pneumococcal disease. Even when a prime-boost regimen containing both conjugated and polysaccharide pneumococcal vaccine is used, only 13% of the immunized HIV patients are high responders at week 96. Recent data indicate that TLR9-agonists have excellent vaccine adjuvant potential and are safe to use in immunocompetent as well as immunocompromised individuals. The aim of this study is to evaluate the qualitative and quantitative i****mmune** **response to pneumococcal vaccination with or without CpG7909 in HIV-infected adults.****

*Primary efficacy parameter:* 1) Quantitative measurement of specific anticapsular antibodies (7 serotypes)

*Secondary efficacy parameters* 1) Functional activity of specific anticapsular antibodies (pneumococcal serotypes 6B, 14, 19F and 23F) 2) Safety/Tolerability measured by: Adverse events (AEs), Serious adverse events (SAEs), Laboratory tests (hematology, clinical chemistry i.e. viral load (HIV RNA) and CD4-count), Physical examination 3) pneumococcal colonization

# **APPENDIX LIST**

****1.**** Investigator’s brochure

2. ****Common toxicity criteria****

3. CIOMS form

4. Participant diary

# SCHEDULE OF ASSESSMENTS/PROCEDURES

| **Assessments/ Procedures** | **Baseline** | **Vaccination** | | | **Follow-up** | | | |
| --- | --- | --- | --- | --- | --- | --- | --- | --- |
| **Study Day** | **Predose** | **Day 0 ^(1)^** | **Day 90 ± 14 ^(2)^** | **Day 270 ± 30 ^(2)^** | **Day 90 ± 14 ^(3)^** | **Day 270 ± 30 ^(4)^** | **Day 300 ± 30 ^(4)^** | |
| Written Informed Consent | X |  |  |  |  |  |  | |
| Inclusion/Exclusion Criteria | **X** |  |  |  |  |  |  | |
| Medical History and Demographics | **X** |  |  |  |  |  |  | |
| Pregnancy Test **^(5)^** | **X** |  | **X** | **X** |  |  |  | |
| Randomization | **X** |  |  |  |  |  |  | |
| Conjugate pneumococcal vaccine ± CPG 7909 **^(6)^** |  | **X** | **X** |  |  |  |  | |
| Polysaccharide pneumococcal vaccine ± CPG 7909 **^(6)^** |  |  |  | **X** |  |  |  |  |
| **Safety** |  |  |  |  |  |  |  | |
| Physical Examination |  | **X^(7)^** | **X^(7)^** | **X^(7)^** | **X^(8)^** | **X^(8)^** | **X^(8)^** | |
| Bloodpressure and Heartrate |  | **X^(7)^** | **X^(7)^** | **X^(7)^** | **X** | **X** | **X** | |
| Lab Safety Tests**^(9, 10)^** | **X** |  |  |  | **X** | **X** | **X** | |
| HIV Control Parameters**^(11)^** | **X** |  |  |  | **X** | **X** | **X** | |
| Adverse Events**^(12)^** |  | **X** | **X** | **X** | **X** | **X** | **X** | |
| **Efficacy** |  |  |  |  |  |  |  | |
| Functional antibody response | **X** |  |  |  | **X** | X | X | |
| Quantitative antibody response | **X** |  |  |  | **X** | **X** | **X** | |
| Oropharyngeal swap | **X** |  |  |  |  | **X** | **X** | |
| Immunologic markers **^(13)^** | **X** |  |  |  | **X** | **X** | **X** | |

1. Day 0 vaccination is conducted after all baseline assessments/procedures are made.
2. Day 90 follow-up and vaccination can vary by 14 days
3. Follow-up is done before proceeding to 2^nd^ vaccination
4. Follow-up day 270 and day 300 is allowed to vary by 30 days.
5. Women of childbearing potential only.
6. According to randomization.
7. Participants asked to stay in for at least ½ hour post-vaccination. A physical examination incl. BP and HR will be made before discharge.
8. Physical examination will be made on participants request or if the investigator finds it relevant.
9. Complete bloodcount, serum chemistry and liver parameters
10. If a participant withdraws prematurely, lab safety tests must be done within approximately one week of participant withdrawal
11. Viral load (HIV RNA) and CD4-count
12. Intensity of adverse events graded according to common toxicity criteria (CTC version 2.0).
13. Immunologic markers (i.e. sCD163) that may predict antibody response and cellular immune response.

# REFERENCE LIST

1. McEllistrem, M. C. *et al*. Recurrent invasive pneumococcal disease in individuals with human immunodeficiency virus infection. *J. Infect. Dis.* **185**, 1364-1368 (2002).

2. Nuorti, J. P. *et al*. Epidemiologic relation between HIV and invasive pneumococcal disease in San Francisco County, California. *Ann. Intern. Med.* **132**, 182-190 (2000).

3. Redd, S. C. *et al*. The role of human immunodeficiency virus infection in pneumococcal bacteremia in San Francisco residents. *J. Infect. Dis.* **162**, 1012-1017 (1990).

4. Barry, P. M. *et al*. Invasive pneumococcal disease in a cohort of HIV-infected adults: incidence and risk factors, 1990-2003. *AIDS* **20**, 437-444 (2006).

5. Grau, I. *et al*. Epidemiologic changes in bacteremic pneumococcal disease in patients with human immunodeficiency virus in the era of highly active antiretroviral therapy. *Arch. Intern. Med.* **165**, 1533-1540 (2005).

6. Feikin, D. R., Feldman, C., Schuchat, A. & Janoff, E. N. Global strategies to prevent bacterial pneumonia in adults with HIV disease. *Lancet Infect. Dis.* **4**, 445-455 (2004).

7. Feikin, D. R. *et al*. Specificity of the antibody response to the pneumococcal polysaccharide and conjugate vaccines in human immunodeficiency virus-infected adults. *Clin. Diagn. Lab. Immunol.* **11**, 137-141 (2004).

8. Rodriguez-Barradas, M. C. *et al*. IgG antibody to pneumococcal capsular polysaccharide in human immunodeficiency virus-infected subjects: persistence of antibody in responders, revaccination in nonresponders, and relationship of immunoglobulin allotype to response. *J. Infect. Dis.* **173**, 1347-1353 (1996).

9. Rodriguez-Barradas, M. C. *et al*. Response of human immunodeficiency virus-infected patients receiving highly active antiretroviral therapy to vaccination with 23-valent pneumococcal polysaccharide vaccine. *Clin. Infect. Dis.* **37**, 438-447 (2003).

10. Kroon, F. P., van Dissel, J. T., Ravensbergen, E., Nibbering, P. H. & van Furth, R. Antibodies against pneumococcal polysaccharides after vaccination in HIV-infected individuals: 5-year follow-up of antibody concentrations. *Vaccine* **18**, 524-530 (1999).

11. Nielsen, H., Kvinesdal, B., Benfield, T. L., Lundgren, J. D. & Konradsen, H. B. Rapid loss of specific antibodies after pneumococcal vaccination in patients with human immunodeficiency virus-1 infection. *Scand. J. Infect. Dis.* **30**, 597-601 (1998).

12. Pneumococcal conjugate vaccine for childhood immunization--WHO position paper. *Wkly. Epidemiol. Rec.* **82**, 93-104 (2007).

13. Global Advisory Committee on Vaccine Safety, 29-30 November 2006. *Wkly. Epidemiol. Rec.* **82**, 18-24 (2007).

14. Kroon, F. P., van Dissel, J. T., Ravensbergen, E., Nibbering, P. H. & van Furth, R. Enhanced antibody response to pneumococcal polysaccharide vaccine after prior immunization with conjugate pneumococcal vaccine in HIV-infected adults. *Vaccine* **19**, 886-894 (2000).

15. Abraham-Van Parijs, B. Review of pneumococcal conjugate vaccine in adults: implications on clinical development. *Vaccine* **22**, 1362-1371 (2004).

16. Jackson, L. A. *et al*. Immunogenicity of varying dosages of 7-valent pneumococcal polysaccharide-protein conjugate vaccine in seniors previously vaccinated with 23-valent pneumococcal polysaccharide vaccine. *Vaccine* **25**, 4029-4037 (2007).

17. Jackson, L. A. *et al*. Safety of varying dosages of 7-valent pneumococcal protein conjugate vaccine in seniors previously vaccinated with 23-valent pneumococcal polysaccharide vaccine. *Vaccine* **23**, 3697-3703 (2005).

18. Nielsen, S. V. & Henrichsen, J. Incidence of invasive pneumococcal disease and distribution of capsular types of pneumococci in Denmark, 1989-94. *Epidemiol. Infect.* **117**, 411-416 (1996).

19. Lockhart, S. P., Hackell, J. G. & Fritzell, B. Pneumococcal conjugate vaccines: emerging clinical information and its implications. *Expert Rev. Vaccines* **5**, 553-564 (2006).

20. Lévy, Y. *et al*. Long-term Immunologenicity of a Prime-boost StrategyCombining a 7-Valent Pneumococcal Conjugate Vaccinefollowed by a 23-Valent Pneumococcal PolysaccharideVaccine vs PPV Alone: ANRS 114 Pneumovac Study. *CROI* **#866** (2007).

21. Abzug, M. J. *et al*. Immunogenicity, safety, and predictors of response after a pneumococcal conjugate and pneumococcal polysaccharide vaccine series in human immunodeficiency virus-infected children receiving highly active antiretroviral therapy. *Pediatr. Infect. Dis. J.* **25**, 920-929 (2006).

22. Petrovsky, N. & Aguilar, J. C. Vaccine adjuvants: current state and future trends. *Immunol. Cell Biol.* **82**, 488-496 (2004).

23. Krieg, A. M. Therapeutic potential of Toll-like receptor 9 activation. *Nat. Rev. Drug Discov.* **5**, 471-484 (2006).

24. Cooper, C. L. *et al*. CPG 7909 adjuvant improves hepatitis B virus vaccine seroprotection in antiretroviral-treated HIV-infected adults. *AIDS* **19**, 1473-1479 (2005).

25. Creticos, P. S. *et al*. Immunotherapy with a ragweed-toll-like receptor 9 agonist vaccine for allergic rhinitis. *N. Engl. J. Med.* **355**, 1445-1455 (2006).

26.

27. Abadi, J. *et al*. Human antibodies elicited by a pneumococcal vaccine express idiotypic determinants indicative of V(H)3 gene segment usage. *J. Infect. Dis.* **178**, 707-716 (1998).

28. Manegold, C., Leichman, G. & Gravenor, D. Addition of PF-3512676 (CpG 7909) to a Taxane/Platinum regimen for first-line treatment of unresectable non-small cell lung cancer (NSCLC) improves objective response — Phase II clinical trial. *Eur. J. Cancer* **3 (Suppl.)**, 326 A1131 (2005).

29. Chu, R. S., McCool, T., Greenspan, N. S., Schreiber, J. R. & Harding, C. V. CpG oligodeoxynucleotides act as adjuvants for pneumococcal polysaccharide-protein conjugate vaccines and enhance antipolysaccharide immunoglobulin G2a (IgG2a) and IgG3 antibodies. *Infect. Immun.* **68**, 1450-1456 (2000).

30. von Hunolstein, C. *et al*. The adjuvant effect of synthetic oligodeoxynucleotide containing CpG motif converts the anti-Haemophilus influenzae type b glycoconjugates into efficient anti-polysaccharide and anti-carrier polyvalent vaccines. *Vaccine* **19**, 3058-3066 (2001).

31. Kovarik, J. *et al*. Adjuvant effects of CpG oligodeoxynucleotides on responses against T-independent type 2 antigens. *Immunology* **102**, 67-76 (2001).

32. Sen, G., Chen, Q. & Snapper, C. M. Immunization of aged mice with a pneumococcal conjugate vaccine combined with an unmethylated CpG-containing oligodeoxynucleotide restores defective immunoglobulin G antipolysaccharide responses and specific CD4+-T-cell priming to young adult levels. *Infect. Immun.* **74**, 2177-2186 (2006).

33. Equils, O. *et al*. Toll-like receptor 2 (TLR2) and TLR9 signaling results in HIV-long terminal repeat trans-activation and HIV replication in HIV-1 transgenic mouse spleen cells: implications of simultaneous activation of TLRs on HIV replication. *J. Immunol.* **170**, 5159-5164 (2003).

34. Agrawal, S. & Martin, R. R. Was induction of HIV-1 through TLR9? *J. Immunol.* **171**, 1621; author reply 1621-2 (2003).

35. Gurney, K. B., Colantonio, A. D., Blom, B., Spits, H. & Uittenbogaart, C. H. Endogenous IFN-alpha production by plasmacytoid dendritic cells exerts an antiviral effect on thymic HIV-1 infection. *J. Immunol.* **173**, 7269-7276 (2004).

36. Schlaepfer, E. *et al*. CpG oligodeoxynucleotides block human immunodeficiency virus type 1 replication in human lymphoid tissue infected ex vivo. *J. Virol.* **78**, 12344-12354 (2004).

37. O'Brien, W. A. *et al*. Human immunodeficiency virus-type 1 replication can be increased in peripheral blood of seropositive patients after influenza vaccination. *Blood* **86**, 1082-1089 (1995).

38. Stanley, S. K. *et al*. Effect of immunization with a common recall antigen on viral expression in patients infected with human immunodeficiency virus type 1. *N. Engl. J. Med.* **334**, 1222-1230 (1996).

39. Ortigao-de-Sampaio, M. B. *et al*. Increase in plasma viral load after oral cholera immunization of HIV-infected subjects. *AIDS* **12**, F145-50 (1998).

40. Sulkowski, M. S. *et al*. The effect of acute infectious illnesses on plasma human immunodeficiency virus (HIV) type 1 load and the expression of serologic markers of immune activation among HIV-infected adults. *J. Infect. Dis.* **178**, 1642-1648 (1998).

41. Delaugerre, C. *et al*. Increase of HIV-1 pro-viral DNA per million peripheral blood mononuclear cells in patients with advanced HIV disease (CD4<200 cells/mm3) receiving interleukin 2 combined with HAART versus HAART alone (ANRS-082 trial). *Antivir Ther.* **8**, 233-237 (2003).

42. Lee, P. K., Kieffer, T. L., Siliciano, R. F. & Nettles, R. E. HIV-1 viral load blips are of limited clinical significance. *J. Antimicrob. Chemother.* **57**, 803-805 (2006).

43. Di Mascio, M. *et al*. Viral blip dynamics during highly active antiretroviral therapy. *J. Virol.* **77**, 12165-12172 (2003).

44. Nettles, R. E. *et al*. Intermittent HIV-1 viremia (Blips) and drug resistance in patients receiving HAART. *JAMA* **293**, 817-829 (2005).

45. Pett, S. L. *et al*. Evaluation of Subcutaneous Proleukin (interleukin-2) in a Randomized International Trial (ESPRIT): geographical and gender differences in the baseline characteristics of participants. *HIV. Clin. Trials* **7**, 70-85 (2006).

46. The ESPRIT Research Group *et al*. Predictors of CD4 count change over 8 months of follow up in HIV-1-infected patients with a CD4 count>or=300 cells/microL who were assigned to 7.5 MIU interleukin-2. *HIV. Med.* **8**, 112-123 (2007).

47. Einhaupl, K. ESPRIT study design and outcomes--a critical appraisal. *Curr. Med. Res. Opin.* **23**, 271-273 (2007).

48. Martinez, J. E., Clutterbuck, E. A., Li, H., Romero-Steiner, S. & Carlone, G. M. Evaluation of multiplex flow cytometric opsonophagocytic assays for determination of functional anticapsular antibodies to Streptococcus pneumoniae. *Clin. Vaccine Immunol.* **13**, 459-466 (2006).

49. Frazao, N. *et al*. Effect of the seven-valent conjugate pneumococcal vaccine on carriage and drug resistance of Streptococcus pneumoniae in healthy children attending day-care centers in Lisbon. *Pediatr. Infect. Dis. J.* **24**, 243-252 (2005).

50. Moher, D., Jones, A., Lepage, L. & CONSORT Group (Consolitdated Standards for Reporting of Trials). Use of the CONSORT statement and quality of reports of randomized trials: a comparative before-and-after evaluation. *JAMA* **285**, 1992-1995 (2001).

51. Madhi, S. A., Kuwanda, L., Cutland, C. & Klugman, K. P. The impact of a 9-valent pneumococcal conjugate vaccine on the public health burden of pneumonia in HIV-infected and -uninfected children. *Clin. Infect. Dis.* **40**, 1511-1518 (2005).

52. Madhi, S. A. *et al*. Quantitative and qualitative antibody response to pneumococcal conjugate vaccine among African human immunodeficiency virus-infected and uninfected children. *Pediatr. Infect. Dis. J.* **24**, 410-416 (2005).

53. Klugman, K. P. *et al*. A trial of a 9-valent pneumococcal conjugate vaccine in children with and those without HIV infection. *N. Engl. J. Med.* **349**, 1341-1348 (2003).

54. Kamchaisatian, W., Wanwatsuntikul, W., Sleasman, J. W. & Tangsinmankong, N. Validation of current joint American Academy of Allergy, Asthma & Immunology and American College of Allergy, Asthma and Immunology guidelines for antibody response to the 23-valent pneumococcal vaccine using a population of HIV-infected children. *J. Allergy Clin. Immunol.* **118**, 1336-1341 (2006).

55. Feikin, D. R. *et al*. Randomized trial of the quantitative and functional antibody responses to a 7-valent pneumococcal conjugate vaccine and/or 23-valent polysaccharide vaccine among HIV-infected adults. *Vaccine* **20**, 545-553 (2001).
